# Sleep Loss Drives Brain Region-Specific and Cell Type-Specific Alterations in Ribosome-Associated Transcripts Involved in Synaptic Plasticity and Cellular Timekeeping

**DOI:** 10.1523/JNEUROSCI.1883-20.2021

**Published:** 2021-06-23

**Authors:** Carlos Puentes-Mestril, James Delorme, Lijing Wang, Marcus Donnelly, Donald Popke, Sha Jiang, Sara J. Aton

**Affiliations:** Department of Molecular, Cellular, and Developmental Biology, University of Michigan, Ann Arbor, Michigan 48019

**Keywords:** circadian rhythms, interneuron, plasticity, principal neurons, sleep deprivation, translation

## Abstract

Sleep and sleep loss are thought to impact synaptic plasticity, and recent studies have shown that sleep and sleep deprivation (SD) differentially affect gene transcription and protein translation in the mammalian forebrain. However, much less is known regarding how sleep and SD affect these processes in different microcircuit elements within the hippocampus and neocortex, for example, in inhibitory versus excitatory neurons. Here, we use translating ribosome affinity purification (TRAP) and *in situ* hybridization to characterize the effects of sleep versus SD on abundance of ribosome-associated transcripts in Camk2a-expressing (Camk2a^+^) pyramidal neurons and parvalbumin-expressing (PV^+^) interneurons in the hippocampus and neocortex of male mice. We find that while both Camk2a^+^ neurons and PV^+^ interneurons in neocortex show concurrent SD-driven increases in ribosome-associated transcripts for activity-regulated effectors of plasticity and transcriptional regulation, these transcripts are minimally affected by SD in hippocampus. Similarly, we find that while SD alters several ribosome-associated transcripts involved in cellular timekeeping in neocortical Camk2a^+^ and PV^+^ neurons, effects on circadian clock transcripts in hippocampus are minimal, and restricted to Camk2a^+^ neurons. Taken together, our results indicate that SD effects on transcripts associated with translating ribosomes are both cell type-specific and brain region-specific, and that these effects are substantially more pronounced in the neocortex than the hippocampus. We conclude that SD-driven alterations in the strength of synapses, excitatory-inhibitory (E-I) balance, and cellular timekeeping are likely more heterogeneous than previously appreciated.

**SIGNIFICANCE STATEMENT** Sleep loss-driven changes in transcript and protein abundance have been used as a means to better understand the function of sleep for the brain. Here, we use translating ribosome affinity purification (TRAP) to characterize changes in abundance of ribosome-associated transcripts in excitatory and inhibitory neurons in mouse hippocampus and neocortex after a brief period of sleep or sleep loss. We show that these changes are not uniform, but are generally more pronounced in excitatory neurons than inhibitory neurons, and more pronounced in neocortex than in hippocampus.

## Introduction

Sleep is essential for optimal brain function, but the underlying biological mechanisms are largely unknown. Prior work aimed at addressing this question has used molecular profiling of mRNA and protein abundance, in a number of brain areas, to characterize changes caused by experimental sleep deprivation (SD; Cirelli et al., 2004; Mackiewicz et al., 2007; Poirrier et al., 2008; Vecsey et al., 2012; Noya et al., 2019). Transcriptomic changes reported after SD in the brain have led to specific hypotheses regarding the biological underpinnings of cognitive disruptions associated with sleep loss (Dinges et al., 1997; Belenky et al., 2003; Mednick et al., 2003; Stickgold, 2005). For example, the synaptic homeostasis hypothesis (Tononi and Cirelli, 2006) proposes that synapses throughout the brain are strengthened during periods of wake and weakened during periods of sleep. The proposal of this hypothesis was initially based on results from transcriptomic studies in mice, showing higher expression of both immediate early genes (IEGs) and several other genes involved in synaptic plasticity after periods of SD versus sleep (Cirelli et al., 1996, 2004; Cirelli and Tononi, 2000; Havekes and Aton, 2020).

However, there may be more heterogeneity in responses to SD across the brain than previously thought. For example, SD-driven transcript changes may vary between different brain structures (Terao et al., 2006; Mackiewicz et al., 2007; Vecsey et al., 2012). We have recently shown that while SD increases expression of the plasticity-mediating IEG *Arc* and Arc protein abundance in neocortical areas (e.g., primary somatosensory cortex; S1), it simultaneously decreases *de novo* synthesis of Arc in the hippocampal dentate gyrus (DG). Indeed, recent data have suggested that SD could differentially impact neuronal activity and dendritic spine density in hippocampal versus neocortical structures (McDermott et al., 2003; Vyazovskiy et al., 2009; Havekes et al., 2016; de Vivo et al., 2017; Ognjanovski et al., 2018; Raven et al., 2019; Havekes and Aton, 2020). Because cognitive processes reliant on the hippocampus, such as episodic memory consolidation (Saletin and Walker, 2012; Havekes and Abel, 2017), seem particularly susceptible to disruption by SD, a critical unanswered question is whether SD differentially impacts network activity and plasticity in the two structures. Beyond this, within brain structures, there may be heterogeneity in the responses of different neuronal subtypes to SD. For example, within the neocortex, fast-spiking interneurons, or neurons with greater firing rates, appear to have differential firing rate changes across periods of sleep (Vyazovskiy et al., 2009; Clawson et al., 2018). Consistent with this idea, synaptic excitatory-inhibitory (E-I) balance was recently shown to vary in neocortex over the course of the day in a sleep-dependent manner (Bridi et al., 2020). Moreover, while most neocortical neurons fire at lower rates during slow wave sleep (SWS) versus wake, some subclasses of neocortical neurons are selectively sleep-active (Gerashchenko et al., 2008).

Here we aimed to better characterize brain region-specific and cell type-specific changes evoked in the nervous system during SD. We used cell type-specific translating ribosome affinity purification (TRAP; Sanz et al., 2019) to profile SD-mediated changes in ribosome-associated mRNAs in two prominent hippocampal and neocortical cell types, Camk2a-expressing (Camk2a^+^) pyramidal neurons and parvalbumin-expressing (PV^+^) interneurons. Because interactions between these two cell types are critical for mediating state-dependent sensory plasticity and memory consolidation (Aton et al., 2013; Kuhlman et al., 2013; Ognjanovski et al., 2017, 2018), we characterized SD-driven changes in ribosome-associated transcripts encoding transcription-regulating IEGs, plasticity effector proteins, and circadian clock components in these two cell types. We find that SD generally causes more modest changes to these transcripts in hippocampal versus neocortical circuits, and in PV^+^ interneurons versus Camk2a^+^ neurons. Together our data suggest that the effects of SD on the brain are more heterogeneous than previously thought, and indicate region-dependent and cell type-dependent differences in SD's impact which may have important implications for brain function.

## Materials and Methods

### 

#### Mouse handling and husbandry

**Figure 1.**
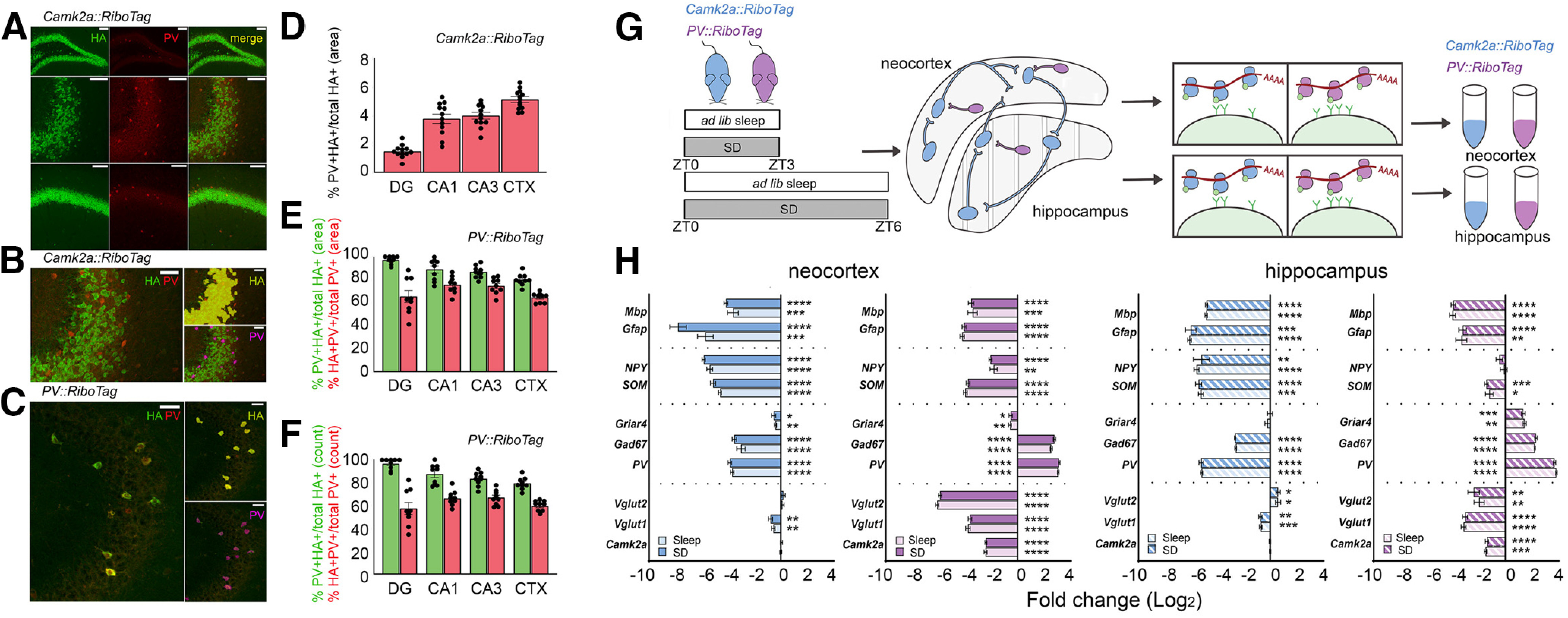
Validation and experimental design for TRAP. ***A***, Antibody staining for Rpl22-HA (green) and PV (red) is shown for dorsal hippocampal subregions DG (top), CA3 (middle), and CA1 (bottom) of a *Camk2a::RiboTag* mouse. Scale bars: 100 µm. ***B***, Example of automated protocol used for *Camk2a::RiboTag* mice to quantify non-specific expression (i.e., overlap of PV^+^ with HA^+^ expression regions; quantified in ***D*** within the pyramidal cell layer of CA3). Areas of overlapping fluorescence were determined using automated detection of HA^+^ (green fluorescence, labeled in yellow) and PV^+^ (red fluorescence, labeled in magenta) areas. Scale bars: 50 µm. ***C***, HA expression in PV^+^ interneurons was validated with immunohistochemistry in *PV::RiboTag* mice, using the method described in ***B***. ***D***, Areas of PV^+^ and HA^+^ overlap (as a proportion of total HA^+^ area) in *Camk2a::RiboTag* sections were minimal. ***E***, ***F***, HA^+^ and PV^+^ overlapping expression presented as a proportion of total PV^+^ or HA^+^ cell counts (red and green, respectively; ***E***) and total PV^+^ or HA^+^ area (red and green, respectively, ***F***) in *PV::RiboTag* sections. ***G***, Experimental design for cell type-specific ribosomal profiling. *Camk2a::RiboTag* (blue) and *PV::RiboTag* (violet) transgenic mice were killed after a 3- or 6-h period of *ad libitum* sleep (sleep) or SD starting at lights on (ZT0). Ribosome-associated mRNAs were affinity purified from hippocampus and neocortex separately. ***H***, Enrichment of markers for glia (*Mbp*, *Gfap*), non-PV^+^ inhibitory neurons (*NPY*, *SOM*), PV^+^ neurons (*Griar4*, *Gad67*, *Pvalb*), and excitatory neurons (*Vglut1*, *Vglut2*, *Camk2a*) calculated as ΔΔCT between affinity purified (RiboTag) mRNA and input mRNA from neocortex or hippocampus. Data presented as log(2) transformed fold changes and are shown separately for SD and sleep conditions. Gene expression was normalized to housekeeping gene pairs according to their respective condition (see Table 1). Values indicate mean ± SEM with propagated error; *p < 0.05, **p < 0.01, ***p < 0.001, ****p < 0.0001, one sample *t* test against a hypothetical value of 0.

**Figure 2.**
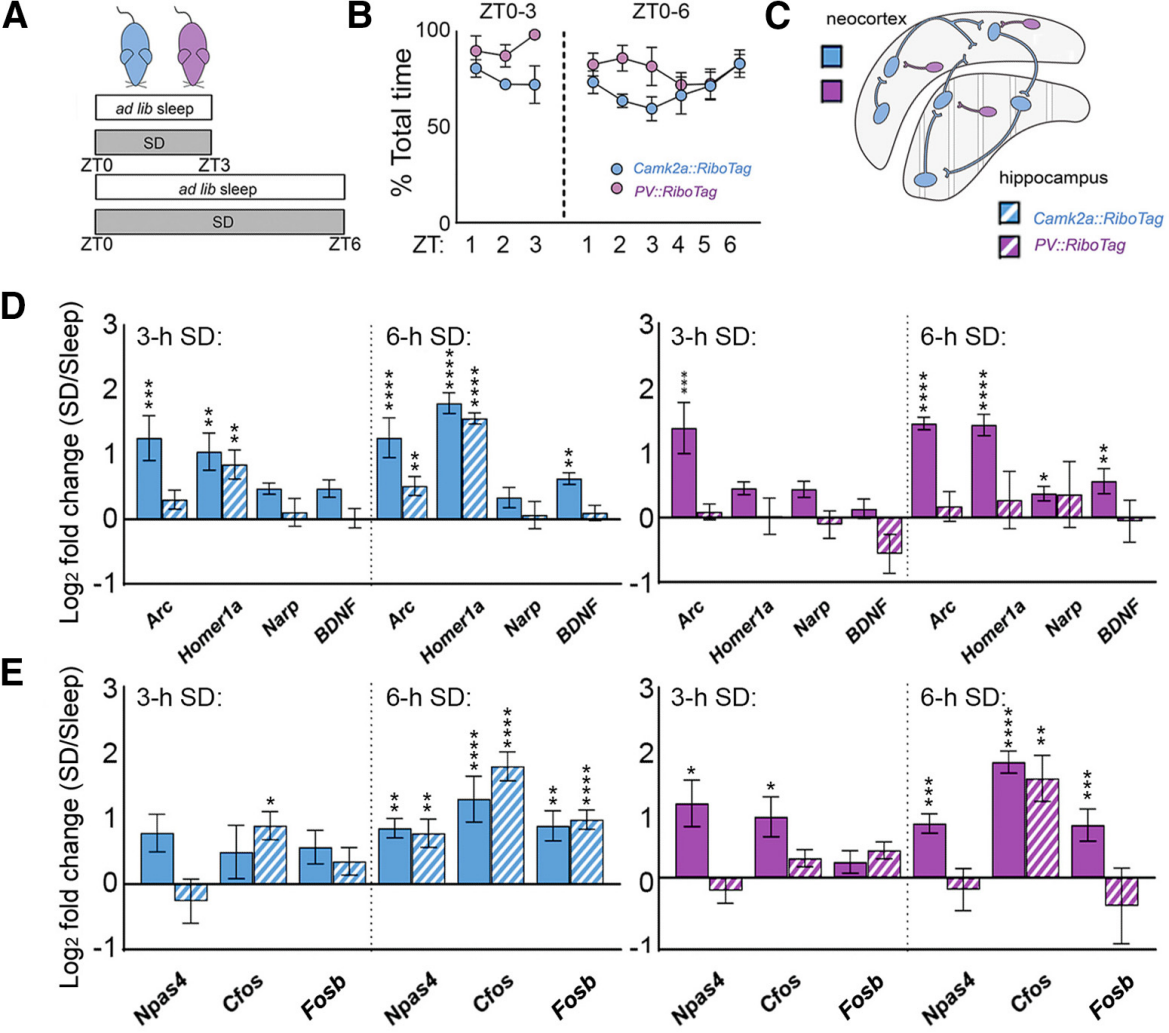
SD increases ribosome-associated plasticity effector transcripts and immediate-early transcription regulators in a cell type-specific and region-specific manner. ***A***, Experimental design for sleep versus SD. ***B***, Sleep amounts for 3- and 6-h *ad libitum* sleep groups, calculated as a percent of total time. ***C***, SD effects on ribosome-associated transcript abundance were quantified separately from neocortex and hippocampus from *Camk2a::RiboTag* (blue) and *PV::RiboTag* (violet) transgenic mice. ***D***, Three-hour SD significantly increased *Arc* and *Homer1a* levels on ribosomes from Camk2a^+^ neocortical (solid) neurons; only *Homer1a* increased in hippocampal (dashed) neurons. Three-hour SD significantly increased *Arc* on ribosomes from PV^+^ interneurons in neocortex; no significant change was observed in the hippocampal PV^+^ interneuron population. *Arc*, *Homer1a*, and *Bdnf* significantly increased after 6-h SD in Camk2a^+^ neocortical neurons; *Arc* and *Homer1a* were increased within the Camk2a^+^ hippocampal population. All effector transcript levels were significantly elevated after 6-h SD in PV^+^ interneurons in neocortex; no significant change was observed in the hippocampal PV^+^ population. Transcript level changes are presented as a log_2_ fold change between SD and *ad libitum* sleep mice. ***E***, Three-hour SD had no significant effect on IEG transcript levels on ribosomes from Camk2a^+^ neocortical (solid) neurons; only *Cfos* increased in hippocampal (dashed) neurons. Three-hour SD significantly increased *Npas4* and *Cfos* on ribosomes from PV^+^ interneurons in neocortex, but did not affect IEG abundance on ribosomes from hippocampal PV^+^ neurons. Six-hour SD significantly increased *Npas4*, C*fos*, and *Fosb* levels in Camk2a^+^ neocortical neurons, Camk2a^+^ hippocampal neurons, and PV^+^ neocortical interneurons. Only *Cfos* significantly increased in the PV^+^ hippocampal population with 6-h SD. Values indicate mean ± SEM with propagated error; *p < 0.05, **p < 0.01, ***p < 0.001, ****p < 0.0001, Holm–Sidak *post hoc* test versus sleep.

**Figure 3.**
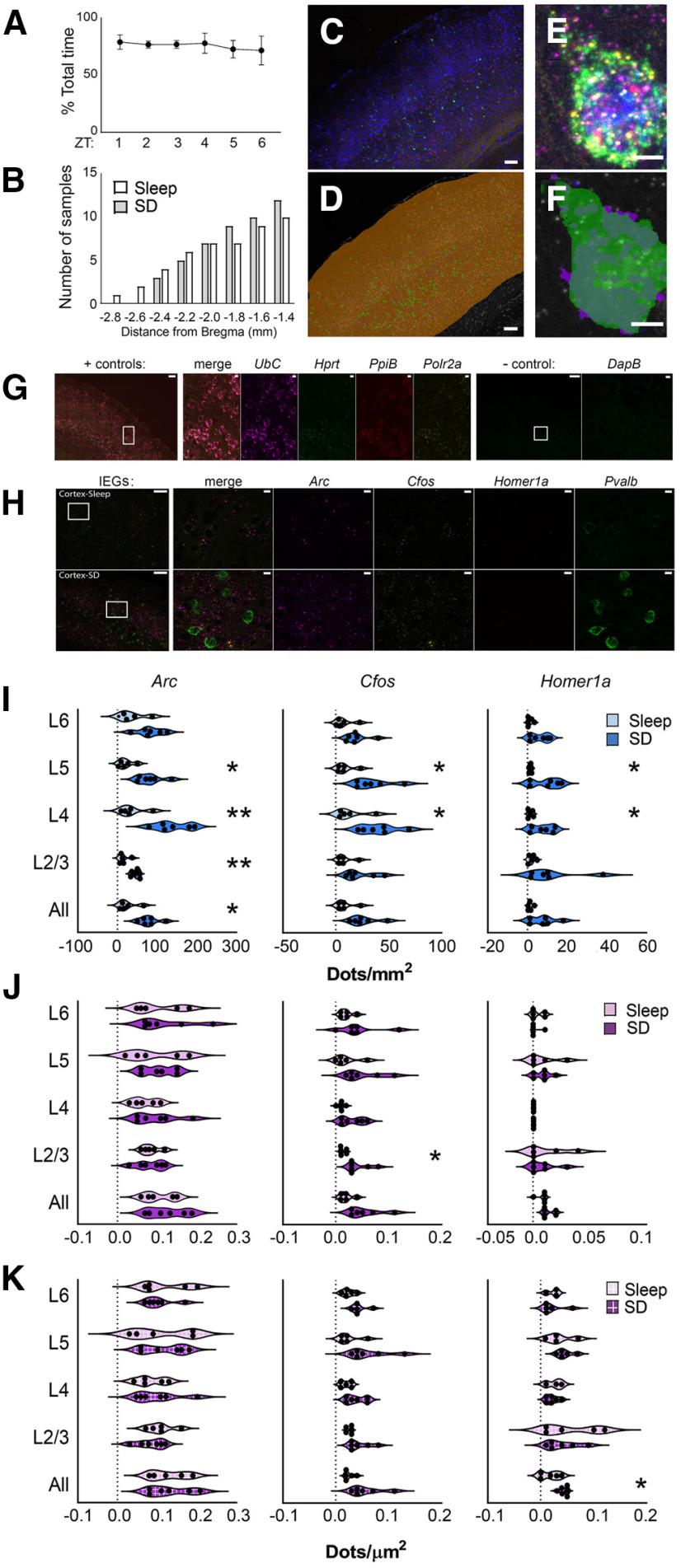
Layer-specific and cell type-specific induction of IEG expression in neocortex after SD. ***A***, Proportion of time spent in *ad libitum* sleep between ZT0 and ZT6 for mice used for fluorescence *in situ* hybridization experiments. ***B***, Cumulative frequency distribution of A/P coordinates (relative to bregma) for brain sections used in analysis. ***C***, A representative image of neocortical *in situ* hybridization for *Arc* (magenta), *Cfos* (yellow), *Homer1a* (red), and *Pvalb* (green). DAPI staining shown in blue. ***D***, Anatomical regions for quantification were demarcated manually (shown in orange). Within these anatomic regions, *Pvalb* (green) fluorescence delineated *Pvalb*+ and non- *Pvalb*+ ROIs. An automated protocol was used to calculate the total fluorescence intensity and area of each ROI (and background). Scale bars: 100 µm. ***E***, Example of IEG and *Pvalb* fluorescence. ***F***, *Pvalb*+ ROI demarcation. Scale bar: 5 µm. ***G***, Representative images showing neocortical riboprobe labeling for ubiquitously expressed (+ control) housekeeping genes *UbC* (magenta), *Hprt1* (green), *PpiB* (red), and *Polr2a* (yellow). Negative control probes targeting DapB mRNA, a gene expressed in *Bacillus subtilis*, shown in the same regions. Inset regions are shown at higher magnification; scale bars: 100 and 10 µm, respectively. ***H***, Representative images of neocortical IEG expression after 6 h of *ad libitum* sleep (*n* = 5 mice) or SD (*n* = 6 mice). Inset regions are shown at higher magnification on right. Scale bars: 100 µm (images) and 10 µm (insets). ***I***, Six-hour SD significantly increased *Arc* expression among non-*Pvalb*+ cells (blue) across neocortex as a whole, and within layers 2/3, 4, and 5, and increased *Cfos* and *Homer1a* expression in layers 4 and 5. ***J***, Six-hour SD significantly increased *Cfos* expression among *Pvalb*+ cells (magenta) in layer 2/3; no other significant changes were observed. ***K***, When analysis was restricted to IEG+ *Pvalb*+ cells (magenta, box pattern), SD significantly increased *Homer1a* levels among *Homer1a*+ *Pvalb*+ cells in whole cortex; no other significant changes were observed. Violin plots show distribution of values for individual mice; *p < 0.05, **p < 0.01, Holm–Sidak *post hoc* test versus sleep.

All animal procedures were approved by the University of Michigan Institutional Animal Care and Use Committee [PHS Animal Welfare Assurance number D16-00072 (A3114-01)]. Animals were maintained on a 12/12 h light/dark cycle (lights on at 8 A.M.) with food and water provided *ad libitum*. Mice expressing Cre recombinase in Camk2a^+^ neurons or PV^+^ interneurons (B6.Cg-Tg(Camk2a-cre)T29-1Stl/J or B6;129P2-*Pvalb^tm1(cre)Arbr^*/J; The Jackson Laboratory) were crossed to RiboTag mice (B6N.129-Rpl22*^tm1.1Psam^/J*; The Jackson Laboratory) to express HA-tagged Rpl22 protein in these neuron populations (Fig. 1*G*). Because of the nature of these crosses, this effectively resulted in roughly half of all cellular Rpl22 ribosomal subunit proteins being tagged with HA. Three- to five-month-old male mice were individually housed one week before all experiments (with beneficial enrichment), and were habituated to handling for 5 d before experiments. Following habituation, and beginning at lights on [zeitgeber time (ZT)0], mice were either allowed *ad libitum* sleep in their home cage or were SD by gentle handling (Durkin and Aton, 2016; Durkin et al., 2017; Delorme et al., 2019; Clawson et al., 2021). For sleeping animals, sleep behavior was visually scored at 5 min or 2 min intervals (for 6- and 3-h SD, respectively; Figs. 2*B*, 3*A*), based on immobility and assumption of characteristic sleep postures. Previous research from our lab has shown that wake time over the final 45 min of the experiment correlates with *Arc* IEG expression in neocortex (Delorme et al., 2019). Thus, to reduce the probability of confounding results from freely-sleeping mice, mice in the sleep groups that spent >60% of the final 45 min of the experiment in wake were excluded from subsequent analysis. All mice were killed with an overdose of pentobarbital (Euthasol) before tissue harvest.

#### Experimental design and statistical analysis

For TRAP experiments, male *Camk2a::RiboTag* and *PV::RiboTag* mice were randomly assigned to 3- or 6-h sleep (*n* = 4 and *n* = 5, respectively) and SD (*n* = 5 and 6, respectively) groups. qPCR data were quantified and statistically compared as described below. For each Ribotag-expressing genotype, 6 male mice were used for HA immunohistochemistry as described below. For *in situ* hybridization, three- to five-month-old male C57Bl/6J mice (The Jackson Laboratory) were randomly assigned to 6-h sleep and SD groups (*n* = 6 each). Image analysis for *in situ* was conducted as described below.

#### TRAP

TRAP was performed using methods described in prior studies (Sanz et al., 2009), with minor modifications. Following 3–6 h of *ad libitum* sleep or SD, animals were euthanized with an overdose of pentobarbitol (Euthasol), their brains extracted, and hippocampi/cortices dissected in dissection buffer [1× HBSS, 2.5 mm HEPES (pH 7.4), 4 mm NaHCO_3_, 35 mm glucose, and 100 µg/ml cycloheximide]. Tissue was then transferred to glass dounce column containing 1 ml of homogenization buffer [10 mm HEPES (pH 7.4), 150 mm KCl, 10 mm MgCl_2_, 2 mm DTT, cOmplete Protease Inhibitor Cocktail (Sigma-Aldrich, 11836170001), 100 U/ml Rnasin Ribonuclease Inhibitors (Promega, N2111), and 100 µg/ml cycloheximide] and manually homogenized on ice. Homogenate was transferred to a 1.5-ml LoBind tubes (Eppendorf) and centrifuged at 1000 × *g* at 4°C for 10 min. Supernatant was then transferred to a new tube, 90 µl of 10% NP-40 was added, and samples were allowed to incubate for 5 min. Following this step, the supernatant was centrifuged at maximum speed for 10 min at 4°C, transferred to a new tube, and mixed with 10 µl of HA-antibody (Abcam, ab9110; Jiang et al., 2015; Shigeoka et al., 2018). Antibody binding proceeded by incubating the homogenate-antibody solution for 1.5 h at 4°C with constant rotation. During the antibody rinse, 200 µl of Protein G Dynabeads (ThermoFisher, 10009D) were washed three times in 0.15 M KCl IP buffer [10 mm HEPES (pH 7.4), 150 mm KCl, 10 mm MgCl_2_, and 1% NP-40] and incubated in supplemented homogenization buffer (10% NP-40). Following this step, supplemented buffer was removed, the homogenate-antibody solution was added directly to the Dynabeads, and the solution incubated for 1 h at 4°C with constant rotation. After incubation, the RNA-bound beads were washed four times in 900 µl of 0.35 M KCl [10 mm HEPES (pH 7.4), 350 mm KCl, 10 mm MgCl_2_, 1% NP-40, 2 mm DTT, 100 U/ml Rnasin Ribonuclease Inhibitors (Promega, N2111), and 100 µg/ml cycloheximide]. During the final wash, beads were placed onto the magnet and moved to room temperature. After removing the supernatant, RNA was eluted by vortexing the beads vigorously in 350-µl RLT (QIAGEN, 79 216). Eluted RNA was purified using RNeasy Micro kit (QIAGEN).

#### Quantitative real-time PCR (qPCR) and stability analysis

qPCR experiments were performed as described previously (Delorme et al., 2019). Briefly, purified mRNA samples were quantified by spectrophotometry (Nanodrop Lite; ThermoFisher) and diluted to equal concentrations. 20–500 ng of mRNA was used to synthesize cDNA using iScript's cDNA Synthesis kit (Bio-Rad), cDNA diluted 1:10 in RNase-free H_2_O, and measured using a CFX96 Real-Time System. Primers were designed for these studies, with the exception of Homer1a, for which sequences were established in a prior study (Mikhail et al., 2017). Primer specificity was confirmed using NIH Primer Blast (for primer sequences, see Extended Data Table 1-1). Three technical replicates were used for each sample. Relative changes in gene expression between sleep and SD were quantified using the ΔΔCT method, and these fold changes are presented on a log scale (log_2_ transformed value equivalent to ΔΔCT) with propagated errors. All statistical analyses were performed on ΔCT values.

**Table 1.** Housekeeping pairs used for RiboTag qPCR conditions

mRNA population	Condition	Region	Gene pair	SD(2^-CT^)/S(2^-CT^)^1^	Fold change^2^
Camk2a	3 h	Cortex	*Actg1*/*Hprt1*	0.98	−1.02
		Hippocampus	*Gapdh*/*Tuba4a*	0.90	−1.11
	6 h	Cortex	*Pgk1*/*Tbp*	0.87	−1.15
		Hippocampus	*Gapdh*/*Tuba4a*	0.92	−1.08
PV	3 h	Cortex	*Actg1*/*Hprt1*	0.82	−1.22
		Hippocampus	*Gapdh*/*Tuba4a*	1.02	1.02
	6 h	Cortex	*Pgk1*/*Tbp*	0.97	−1.03
		Hippocampus	*Gapdh*/*Tuba4a*	1.02	1.02

Change in gene expression presented as ratio^1^ and fold change^2^.

10.1523/JNEUROSCI.1883-20.2021.tab1 Extended Data Table 1-1. Primer sequences for qPCR. Download Table 1-1, DOCX file.

10.1523/JNEUROSCI.1883-20.2021.tab2 Extended Data Table 1-2. Housekeeping gene stability analysis for *Camk2a::RiboTag* qPCR. Methods for stability analysis are described in Materials and Methods. *Genorm automatically calculates the stability measure for the two most stable genes. Download Table 1-2, DOCX file.

10.1523/JNEUROSCI.1883-20.2021.tab3 Extended Data Table 1-3. Housekeeping gene stability analysis for *PV::RiboTag* qPCR. *Genorm automatically calculates the stability measure for the two most stable genes. Download Table 1-3, DOCX file.

Reference (housekeeping) genes for normalization were chosen for each experiment based on three measures: intragroup variability, intergroup variability, and an overall stability measure derived from total variance (Extended Data Tables 1-2, 1-3). Special emphasis was placed on selecting pairs of reference transcripts with countervailing intergroup differences. These measures were calculated using Normfinder (Andersen et al., 2004) and RefFinder (Xie et al., 2012) software. Because Normfinder is better suited for large sample sizes, RefFinder was used to validate Normfinder rankings and ensure genes with low (or opposite-direction) intergroup variability were chosen as housekeeping pairs. Stability measures were calculated for each sleeping condition, region, and mRNA population and repeated for mRNAs purified from *PV::RiboTag* and *Camk2a::Ribotag* mice (Table 1). The arithmetic mean of each housekeeping pair was then used to normalize target gene expression. As a final measure of housekeeping stability, we calculated each pairs' fold change between mice in SD and sleep groups.

#### RNAScope *in situ* hybridization

**Figure 4.**
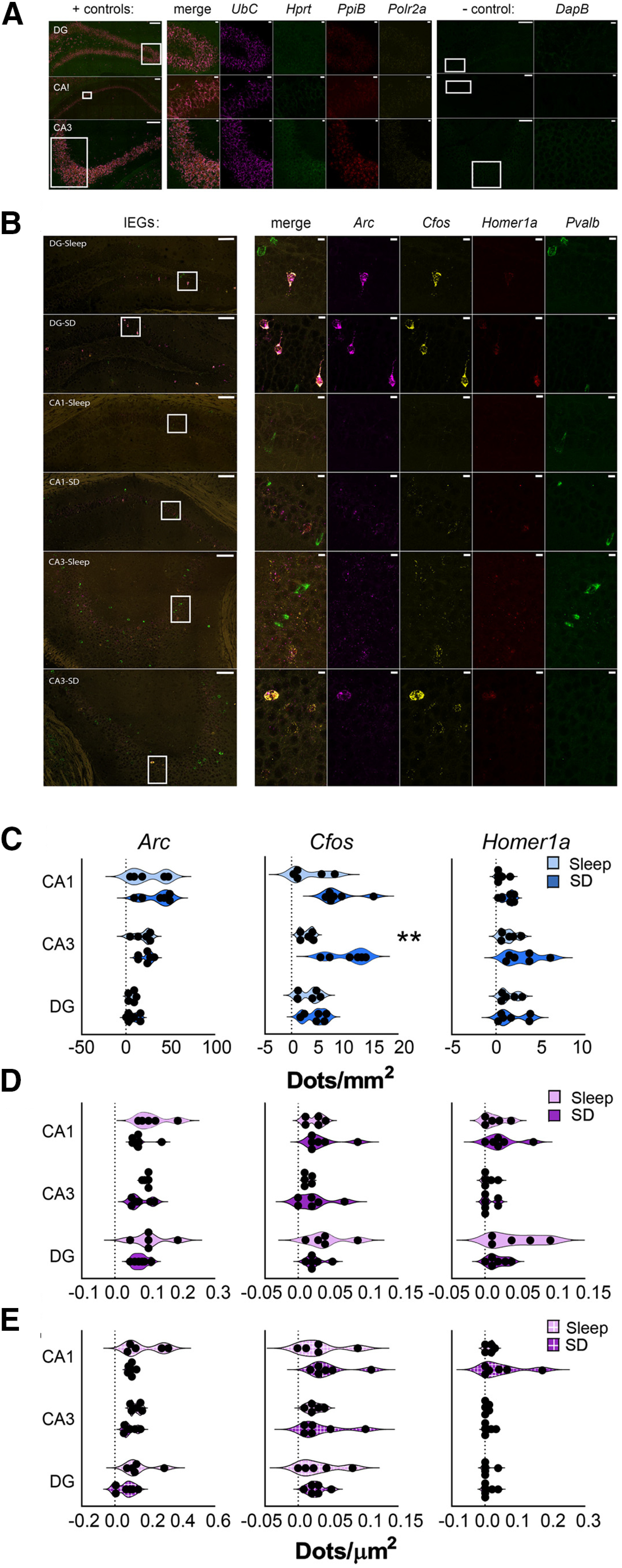
Cell type-specific and region-specific changes in hippocampal IEG expression after SD. ***A***, Representative *in situ* images showing DG, CA1, and CA3 riboprobe labeling for + control and – control genes, as in Figure 3*G*. Inset regions are shown at higher magnification; scale bars: 100 and 10 µm, respectively. ***B***, Representative images of IEG expression after 6 h of *ad libitum* sleep (*n* = 5 mice) or SD (*n* = 6 mice). *In situ* hybridization is shown for *Arc* (magenta), *Cfos* (yellow), *Homer1a* (red), and *Pvalb* (green). Inset regions are shown at higher magnification on right. Scale bars: 100 µm (images) and 10 µm (insets). ***C***, Six-hour SD significantly increased *Cfos* expression among non-*Pvalb*+ (blue) cells in CA3; no other significant changes observed. ***D***, ***E***, No significant changes were observed within DG, CA3, or CA1 in *Pvalb*+ cells (magenta; ***D***) or IEG+ *Pvalb*+ cells (magenta, box pattern; ***E***). Violin plots show distribution of individual subjects; **p < 0.01, Holm–Sidak *post hoc* test versus sleep.

Fluorescent *in situ* hybridization was performed on 14-µm coronal sections taken from fixed-frozen brains of sleep (*n* = 6) and SD (*n* = 6) mice. Section coordinates (1–3.0 mm lateral, −1.4 to −2.8 mm posterior to bregma) were similarly distributed between sleep and SD conditions (Fig. 3*B*). The RNAScope Multiplex Fluorescent Reagent kit v2 with 4-plex ancillary kit was used to label *Arc*, *Homer1a*, *Cfos*, and *Pvalb* transcripts (Fig. 3*C–F*). Before probe incubation, slices were pretreated with hydrogen peroxide (10 min, room temperature), Target Retrieval reagent (5 min 99°C), and RNscope Protease III (30 min, 40°C). Slices were incubated with custom-synthesized *Arc* (20 bp, target region: 23–1066, 316911-C3, Advanced Cell Diagnostics), *Cfos* (20 bp, target region: 407–1427, 316921-C1, Advanced Cell Diagnostics), *Homer1a* (6 bp, target region: 1301-1887m 433941-C2, Advanced Cell Diagnostics), and *Pvalb* 16 (16 bp, target region: 2–885, 421931-C4, Advanced Cell Diagnostics). Probes were chosen so as to overlap with regions amplified by qPCR primer pairs (Extended Data Table 1-1). *Arc*, *Cfos*, *Homer1a*, and *Pvalb* were hybridized to Opal Dyes 620 (FP1495001KT, Akoya Biosciences), 570 (FP1488001KT, Akoya Biosciences), 690 (FP1497001KT, Akoya Biosciences), and 520 (FP1487001, Akoya Biosciences), respectively, for visualization. Positive and negative control probes were used in parallel experiments to confirm the specificity of hybridization signals (Fig. 3*G*,*H*, Fig. 4*A,B*).

#### Immunohistochemistry

For immunohistochemical validation of appropriately cell targeted HA expression in RiboTag-expressing mice, *Camk2a::RiboTag* and *PV::RiboTag* mice from sleep (*n* = 6) and SD (*n* = 6) groups were killed and perfused with PBS followed by 4% paraformaldehyde; 50-µm brain sections were blocked with normal goat serum for 2 h and incubated overnight using biotin-conjugated anti-HA (Biolegend 901505, 1:500) and anti-PV (Synaptic Systems 195004, 1:500) antibodies at 4°C. The following day, sections were stained with streptavidin-Alexa Fluor 647 (Biolegend 405237) and Alexa Fluor 555 goat anti-guinea pig IgG H&L (Abcam ab150186). Stained sections were coverslipped in ProLong Gold Antifade Reagent (ThermoFisher, P36930). Fluorescence intensity was used to identify HA-expressing (HA^+^) cells, PV^+^ cells, and overlapping cells within the DG, CA1, CA3, and neocortex. To account for differences in localization and spread of antibody staining, both PV^+^ HA^+^ cells and HA^+^ PV-expressing cells were identified, and overlap was quantified in terms of both cell count and cell area (Fig. 1*A–F*). Quantification was performed using the semi-automated protocol detailed below. Camk2a antibody staining was not used, as widespread diffuse neuropil labeling made accurate cell counting (i.e., detection of immunonegative cell bodies) infeasible.

#### Imaging and quantification

**Figure 5.**
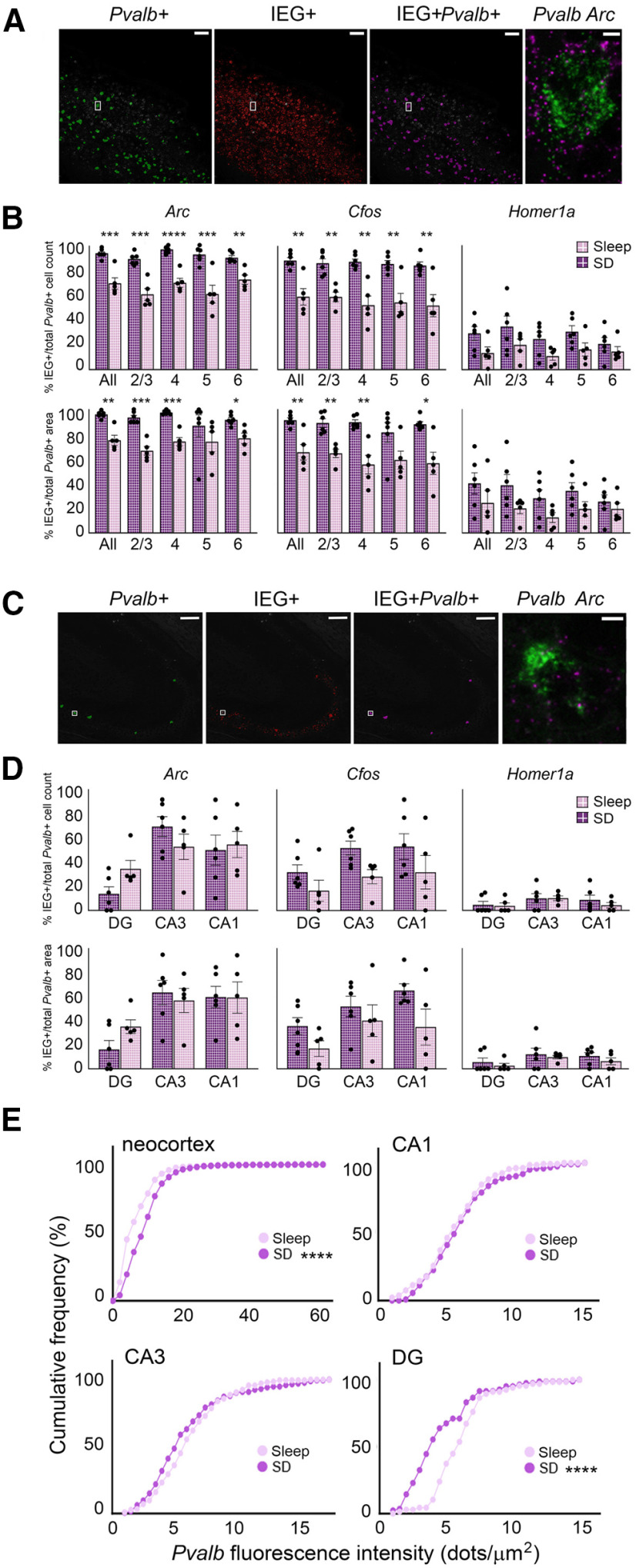
SD increases the proportion of IEG+ *Pvalb*+ interneurons and *Pvalb* expression in neocortex but not hippocampus. ***A***, An automated protocol identified *Pvalb* (green) and IEG (red) *in situ* fluorescence in neocortex; cells with overlapping fluorescence were marked as IEG+*Pvalb*+ (magenta). Inset, *In situ* fluorescence for *Pvalb* (green) and *Arc* (magenta) in a single neuron, shown at right. Total IEG+*Pvalb*+ area was then calculated as the proportion of total *Pvalb*+ area. Scale bars: 100 µm (images) and 5 µm (insets). ***B***, Six-hour SD significantly increased the proportion (top row) and area (bottom row) of *Pvalb*+ cells expressing *Arc* or *Cfos*, but not *Homer1a*, across most neocortical layers. Values indicate mean ± SEM. ***C***, The same method shown in ***A*** identified IEG+ *Pvalb*+ cells within hippocampal subregions DG, CA1, and CA3. Area CA3 shown; scale bars: 100 µm (images) and 5 µm (insets). ***D***, SD had no effect on the proportion (top row) or area (bottom row) of *Pvalb*+ hippocampal cells expressing *Arc*, *Cfos*, or *Homer1a*, Values indicate mean ± SEM. ***E***, Cumulative frequency distributions showing the impact of 6-h SD on *Pvalb* fluorescence intensity in *Pvalb*+ cells of the neocortex and hippocampus. SD significantly increased mean fluorescence intensity of *Pvalb* within *Pvalb*+ cells of the neocortex. SD did not significantly alter *Pvalb* fluorescence intensity among *Pvalb*+ cells in CA3 or CA1 but significantly decreased mean *Pvalb* fluorescence intensity within the DG while having no significant effect on (***B***) CA1 or (***C***) CA3 intensity. *p < 0.05, **p < 0.01, ***p < 0.001, ****p < 0.0001, Holm–Sidak *post hoc* test versus sleep.

RNAScope probe fluorescence signals were captured and stitched using a 40× objective lens on a Leica 3D STED SP8. Immunostained brain sections were imaged on a Leica SP5 laser scanning confocal microscope. Settings were fixed for each imaging session. Fluorescence images were analyzed using MIPAR image analysis software in their raw grayscale format (Sosa et al., 2014). Two images per region (one per hemisphere) were quantified for each animal. Quantification was performed separately for regions CA1, CA3, and DG in dorsal hippocampus, and layers 2/3, 4, 5, and 6 of overlying (i.e., dorsal) neocortical regions (including S1). Total fluorescence dot number and average intensity of a single dot calculated per the recommended guidelines provided by ACDBio (ACDBio, 2017), for *Pvalb*+ and non-*Pvalb*+ regions of interest (ROIs) within granule (DG), pyramidal (CA1, CA3), and cortical layers 1–6 (layers were manually isolated using a freehand tool by a scorer blind to experimental condition). Fluorescence intensity and expression overlap were calculated using a semi-automated protocol curated by blinded scorer. Briefly, a non-local means filter was used to reduce image noise, and an adaptive threshold was used to identify areas >30 µm whose mean pixel intensity was 200% of its surroundings. Identified areas were labeled as IEG+ or *Pvalb*+ and manually edited to refine labeling, select for representative dots, and remove artifacts (manual editing was not used to label any additional IEG expression). Finalized labeling was used to delineate *Pvalb*+ and non-*Pvalb*+ ROIs, select for background area (area in the ROI minus areas of labeled expression), and identify IEG+ *Pvalb*+ cells (Fig. 5). Intensity values from ROIs, background, and selected dots were used to calculate fluorescence dots/area. Average background intensity was calculated as the fluorescence intensity of the selected background area per unit area. The average intensity of a single fluorescent dot was calculated for each transcript as the intensity of manually selected representative dots within the ROI, minus the average background intensity multiplied by the area, divided by the total number of selected dots. Dot intensity values did not differ between sleep and SD mice for specific transcripts. The total fluorescent dot number within each ROI was calculated by subtracting average background intensity from total ROI fluorescence intensity, multiplied by total area, divided by average dot intensity.

## Results

### TRAP-based characterization of ribosome-associated transcripts in Camk2a^+^ and PV^+^ neuronal populations

To quantify how ribosome-associated transcripts in different neuronal populations within the hippocampus and neocortex are affected by sleep loss, we crossed RiboTag transgenic mice (with Cre recombinase-dependent expression of HA-tagged Rpl22 protein) to either Camk2a-Cre or PV-Cre transgenic lines (Sanz et al., 2019). Appropriate cell type-specific expression of Rpl22^HA^ in *Camk2a::RiboTag* and *PV::RiboTag* mice was verified using immunohistochemistry (Fig. 1*A–C*). HA expression was largely circumscribed to the intended cell type. For example, expression of HA in *Camk2a::RiboTag* was appropriately localized to cell bodies in the pyramidal cell layer of hippocampal areas CA1, CA3, and DG (Fig. 1*A*). Expression of HA in non-targeted cell types of the hippocampus (e.g., colabeling for PV in HA^+^ cells in *Camk2a::RiboTag* mice) was minimal (3.6 ± 0.2% on average; Fig. 1*D*). In the hippocampus and neocortex of *PV::RiboTag* mice, 86.7 ± 1.5% and 79.4 ± 1.8% of HA^+^ neurons, respectively, co-expressed PV peptide; similar values for overlap were calculated when either the total area of HA-immunopositive and PV-immunopositive regions within these structures, or immunopositive cell counts, were directly compared (Fig. 1*E*,*F*). We next validated cell type-specificity of ribosome-associated transcripts isolated from transgenic mouse lines. Following a period of *ad libitum* sleep of SD starting at lights on (ZT0), hippocampi and neocortex were dissected, and ribosome-associated mRNAs were isolated (Sanz et al., 2019). We compared abundance of cell type-specific transcripts between RiboTag affinity purified mRNA and Input mRNA from whole hippocampus or neocortex homogenate using qPCR (Fig. 1*G*). Enrichment or de-enrichment of these cell markers was compared with a null hypothetical value of 0 using one-sample *t* tests. We found that ribosomal-associated transcripts from both the neocortex and hippocampus of *Camk2a::RiboTag* mice de-enriched for markers of glial cell types (*Mbp*, *Gfap*), non-PV^+^ inhibitory neurons (*Npy*, *Sst*), PV interneurons (*Gad67*, *Pvalb*), and *Vglut1* relative to input (Fig. 1*H*). Hippocampal enrichment patterns mirrored those of the neocortex with the exception of *Vglut2*, which was significantly enriched relative to Input. Ribosome-associated transcripts from *PV::RiboTag* mice de-enriched for markers of glial (*Mbp*, *Gfap*), non-PV^+^ inhibitory (*Npy*, *Sst*), and excitatory neurons (*Vglut1*, *Vglut2*, *Camk2a*) while enriching for PV^+^ interneuron markers (*Pvalb*, *Gad67*) relative to Input. We made comparisons of cell type-specific transcript enrichment separately for mice which were either allowed *ad libitum* sleep or SD over the first 3 or 6 h after lights on (i.e., from ZT0 to ZT3 or ZT0 to ZT6). No substantial differences in enrichment patterns were observed between sleep and SD mice (not significant (N.S.), Holm–Sidak *post hoc* test). These data confirm the high degree of specificity of TRAP-based profiling for ribosomal transcripts from Camk2a^+^ principal neurons and PV^+^ interneurons.

### SD-driven changes in ribosome-associated plasticity-related mRNAs and activity-dependent transcription regulators vary with cell type and brain structure

We first quantified a subset of transcripts encoding for proteins involved in synaptic plasticity (i.e., plasticity effectors) whose expression levels have been reported previously as altered by SD, *Arc*, *Homer1a*, *Narp*, and *Bdnf* (Cirelli et al., 2004; Maret et al., 2007). Ribosome-associated transcript abundance was first quantified in Camk2a^+^ neocortical and hippocampal neuron populations after 3 h of *ad libitum* sleep (sleep; *n* = 4) or SD (*n* = 5), starting at lights on (ZT0; Fig. 2*A–C*). Consistent with previous findings (Cirelli et al., 2004), 3-h SD significantly increased neocortical *Arc* (*p* < 0.001, Holm–Sidak *post hoc* test) and *Homer1a* (*p* < 0.01; Maret et al., 2007) ribosome-associated mRNA (Fig. 2*D*; Table 2). In contrast, 3-h SD significantly increased *Homer1a* abundance on hippocampal ribosomes (*p* < 0.01), but did not significantly affect *Arc* abundance (N.S., Holm–Sidak *post hoc* test). This is consistent with recent data showing that brief SD results in either no change, or a decrease, in *Arc* expression in various hippocampal subregions (Delorme et al., 2019). Overall patterns of transcript abundance for the plasticity-regulating proteins *Bdnf* and *Narp* followed a similar trend, with unchanged levels in hippocampal Camk2a^+^ neurons (N.S, Holm–Sidak *post hoc* test), and modestly (but not significantly) increased levels in neocortical neurons (*Narp* and *Bdnf*, *N.S*.). After more prolonged (6 h) SD (*n* = 6 mice/group), ribosome-associated *Arc* (*p* < 0.0001), *Homer1a* (*p* < 0.0001), and *Bdnf* (*p* < 0.01) transcripts were all increased in neocortical Camk2a^+^ neurons, whereas *Arc* (*p* < 0.01) and *Homer1a* (*p* < 0.0001) were increased in hippocampal Camk2a^+^ neurons (Fig. 2*D*; Table 2).

**Table 2.** SD-Induced changes in ribosome-associated transcript abundance in *Camk2a::RiboTag* mice

Gene name	Region	Sleep, ΔCT		SD, ΔCT		qPCR *p* value (sleep vs SD)		*n* (sleep)		*n* (SD)	
		3 h	6 h	3 h	6 h	3 h	6 h	3 h	6 h	3 h	6 h
*Arc*	CTX	5.16 ± 0.37	2.20 ± 0.26	3.9 ± 0.19	0.93 ± 0.17	0.0003	<0.0001	4	6	5	6
	HP	4.29 ± 0.07	5.05 ± 0.09	3.98± 0.15	4.53 ± 0.12	0.4663	0.0036	4	6	5	6
*Homer1a*	CTX	5.11 ± 0.29	2.25 ± 0.16	4.053 ± 0.18	0.44 ± 0.04	0.0017	<0.0001	4	6	5	6
	HP	5.97 ± 0.09	5.33 ± 0.74	5.12 ±0.232	3.76 ± 0.04	0.0036	<0.0001	4	6	5	6
*Narp*	CTX	5.34 ± 0.05	2.00 ± 0.10	4.861 ± 0.08	1.66 ± .12	0.1688	0.0902	4	6	5	6
	HP	6.09 ± 0.13	4.932 ± 0.16	5.98 ±0.21	4.86 ± 0.15	0.8637	0.7475	4	6	5	6
*Bdnf*	CTX	5.02 ± 0.12	1.54 ± 0.06	4.542 ± 0.10	0.90 ± 0.07	0.1688	0.0049	4	6	5	6
	HP	3.94 ± 0.03	4.09 ± 0.10	3.92 ± 0.16	3.99 ± 0.07	0.9288	0.7475	4	6	5	6
*Npas4*	CTX	8.42 ± 0.23	4.34 ± 0.12	7.64 ± 0.20	3.50 ± 0.10	0.1370	0.0031	4	6	5	6
	HP	8.61 ± 0.38	8.58 ± 0.18	8.87 ± 0.14	7.80 ± 0.12	0.4478	0.0005	4	6	5	6
*cFos*	CTX	6.17 ± 0.42	3.11 ± 0.31	5.68 ± 0.24	1.81 ± 0.17	0.2664	<0.0001	4	6	5	6
	HP	7.64 ± 0.15	8.32 ± 0.13	6.74 ± 0.20	6.52 ± 0.18	0.0209	<0.0001	4	6	5	6
*FosB*	CTX	5.99 ± 0.12	2.87 ± 0.21	5.42 ± 0.26	1.98 ± 0.10	0.2664	0.0031	4	6	5	6
	HP	6.45 ± 0.09	6.69 ± 0.13	6.10 ± 0.22	5.71 ± 0.07	0.4478	<0.0001	4	6	5	6
*Clock*	CTX	3.95 ± 0.07	0.97 ± 0.08	3.76 ± 0.02	0.93 ± 0.04	0.6176	0.9770	4	6	5	6
	HP	3.45 ± 0.07	3.63 ± 0.06	3.35 ± 0.05	3.56 ± 0.06	0.8666	0.6647	4	6	5	6
*Bmal1*	CTX	4.68 ± 0.11	1.34 ± 0.10	4.71 ± 0.09	1.27 ± 0.07	0.8745	0.9770	4	6	5	6
	HP	4.35 ± 0.09	4.37 ± 0.05	4.39 ± 0.08	4.38 ± 0.06	0.9378	0.9028	4	6	5	6
*Cry1*	CTX	5.90 ± 0.09	2.33 ± 0.08	5.78 ± 0.10	2.24 ± 0.07	0.8434	0.9592	4	6	5	6
	HP	5.88 ± 0.10	6.34 ± 0.10	6.07 ± 0.11	6.10 ± 0.05	0.5022	0.0138	4	6	5	6
*Cry2*	CTX	4.23 ± 0.03	0.36 ± 0.05	4.36 ± 0.11	0.42 ± 0.04	0.8434	0.977	4	6	5	6
	HP	3.80 ± 0.02	3.75 ± 0.04	4.10 ± 0.11	3.95 ± 0.01	0.1126	0.0493	4	6	5	6
*Per1*	CTX	3.90 ± 0.16	0.23 ± 0.10	3.97 ± 0.13	0.19 ± 0.11	0.8745	0.9770	4	6	5	6
	HP	3.62 ± 0.05	3.31 ± 0.03	3.73 ± 0.11	3.34 ± 0.05	0.8666	0.9028	4	6	5	6
*Per2*	CTX	7.43 ± 0.08	3.71 ± 0.18	6.87 ± 0.10	3.38 ± 0.06	0.0012	0.0654	4	6	5	6
	HP	6.62 ± 0.07	6.80 ± 0.07	6.62 ± 0.10	6.56 ± 0.04	0.9744	0.0138	4	6	5	6
*Rev-Erba*	CTX	3.32 ± 0.16	−0.50 ± 0.14	3.48 ± 0.09	0.02 ± 0.06	0.7553	0.0066	4	6	5	6
	HP	4.05 ± 0.09	1.92 ± 0.05	3.91 ± 0.05	2.13 ± 0.03	0.3593	0.7601	4	6	5	6
*Dbp*	CTX	5.29 ± 0.15	2.47 ± 0.08	5.54 ± 0.13	2.85 ± 0.07	0.5635	0.0570	4	6	5	6
	HP	5.33 ± 0.07	4.05 ± 0.24	5.61 ± 0.09	4.03 ± 0.37	0.0583	0.9171	4	6	5	6
*Tef*	CTX	3.47 ± 0.02	0.62 ± 0.07	3.58 ± 0.07	0.74 ± 0.04	0.7553	0.4556	4	6	5	6
	HP	3.14 ± 0.02	2.54 ± 0.05	3.14 ± 0.08	2.33 ± 0.04	0.9679	0.7601	4	6	5	6
*Nfil3*	CTX	6.34 ± 0.14	3.80 ± 0.19	6.29 ± 0.07	3.62 ± 0.07	0.7553	0.4310	4	6	5	6
	HP	6.19 ± 0.07	5.45 ± 0.05	5.94 ± 0.04	4.94 ± 0.07	0.0997	0.083	4	6	5	6
*ec1*	CTX	3.36 ± 0.16	−0.43 ± 0.15	3.18 ± 0.15	−0.77 ± 0.08	0.7553	0.0754	4	6	5	6
	HP	2.74 ± 0.06	1.37 ± 0.05	2.55 ± 0.11	1.21 ± 0.05	0.2666	0.7601	4	6	5	6

We next quantified ribosome-associated transcript abundance in PV^+^ interneuron populations from the neocortex (*n* = 4 mice/group) and hippocampus (*n* = 4 and *n* = 5 mice for sleep and SD; Table 3). Three-hour SD significantly increased *Arc* (*p* < 0.001, Holm–Sidak *post hoc* test) abundance in neocortical PV^+^ interneurons, but had no effect on transcript abundance for plasticity-related proteins in hippocampal PV^+^ interneurons (N.S., Holm–Sidak *post hoc* test). Six-hour SD increased abundance of these transcripts in the neocortical PV^+^ interneuron population (*n* = 5 and *n* = 6 mice for sleep and SD) in a manner similar to the Camk2a^+^ neuronal population (*Arc*, *p* < 0.0001; *Homer1a*, *p* < 0.0001; *Narp*, *p* < 0.05; *Bdnf*, *p* < 0.01). In contrast, 6-h SD caused no significant change in any of the ribosome-associated transcripts' abundance in hippocampal PV^+^ interneurons (*n* = 6 mice/group).

**Table 3.** SD-induced changes in ribosome-associated transcript abundance in *PV::RiboTag* mice

Gene name	Region	Sleep, ΔCT		SD, ΔCT		qPCR *p* value (sleep vs SD)		*n* (sleep)		*n* (SD)	
		3 h	6 h	3 h	6 h	3 h	6 h	3 h	6 h	3 h	6 h
*Arc*	CTX	6.64 ± 0.23	6.87 ± 0.07	5.25 ± 0.43	5.40 ± 0.07	0.0002	<0.0001	4	5	4	6
	HP	6.67 ± 0.09	5.46 ± 0.14	6.58 ± 0.11	5.29 ± 0.19	0.9758	0.8837	4	6	5	6
*Homer1a*	CTX	7.49 ± 0.08	6.71 ± 0.13	7.03 ± 0.10	5.27 ± 0.12	0.3025	<0.0001	4	5	4	6
	HP	9.02 ± 0.23	7.32 ± 0.35	9.00 ± 0.23	7.04 ± 0.27	0.9758	0.8673	4	6	5	6
*Narp*	CTX	8.22 ± 0.08	7.39 ± 0.07	7.77 ± 0.13	7.01 ± 0.09	0.3025	0.0196	4	5	4	6
	HP	8.60 ± 0.15	8.58 ± 0.47	8.71 ± 0.19	8.22 ± 0.21	0.9758	0.8383	4	6	5	6
*Bdnf*	CTX	8.03 ± 0.12	7.36 ± 0.19	7.89 ± 0.14	6.79 ± 0.09	0.6257	0.0014	4	5	4	6
	HP	0.60 ± 0.21	5.38 ± 0.26	1.17 ± 0.27	5.44 ± 0.20	0.2158	0.8907	4	6	5	6
*Npas4*	CTX	9.14 ± 0.43	7.97 ± 0.13	7.96 ± 0.14	7.11 ± 0.10	0.0143	0.0008	4	5	4	6
	HP	9.15 ± 0.20	5.21 ± 0.31	9.36 ± 0.13	5.40 ± 0.12	0.2737	0.6749	4	6	5	6
*cFos*	CTX	7.81 ± 0.10	7.04 ± 0.13	6.85 ± 0.38	5.21 ± 0.13	0.0336	<0.0001	4	5	4	6
	HP	9.81 ± 0.13	7.56 ± 0.32	9.51 ± 0.10	5.98 ± 0.18	0.2081	0.0042	4	6	5	6
*FosB*	CTX	7.93 ± 0.16	7.05 ± 0.17	7.68 ± 0.15	6.22 ± 0.20	0.5072	0.0008	4	5	4	6
	HP	10.86 ± 0.12	6.65 ± 0.45	10.42 ± 0.11	7.10 ± 0.40	0.0814	0.5437	4	6	5	6
*Clock*	CTX	3.58 ± 0.07	3.29 ± 0.09	3.47 ± 0.04	3.04 ± 0.06	0.8513	0.2076	4	5	4	6
	HP	4.11 ± 0.05	4.06 ± 0.58	4.02 ± 0.05	2.68 ± 0.08	0.9716	0.9770	4	6	5	6
*Bmal1*	CTX	5.42 ± 0.09	4.51 ± 0.08	5.25 ± 0.07	4.51 ± 0.04	0.6893	0.9848	4	5	4	6
	HP	5.86 ± 0.09	3.71 ± 0.56	5.93 ± 0.09	5.59 ± 0.12	0.9716	0.9770	4	6	5	6
*Cry1*	CTX	8.57 ± 0.08	4.45 ± 0.03	8.63 ± 0.13	4.51 ± 0.05	0.8942	0.9436	4	5	4	6
	HP	6.24 ± 0.04	3.98 ± 0.60	6.15 ± 0.07	4.22 ± 0.23	0.9716	0.9099	4	6	5	6
*Cry2*	CTX	6.37 ± 0.05	3.12 ± 0.07	6.42 ± 0.09	3.09 ± 0.06	0.8942	0.979	4	5	4	6
	HP	5.18 ± 0.05	3.63 ± 0.61	5.09 ± 0.04	2.82 ± 0.07	0.9716	0.9994	4	6	5	6
*Per1*	CTX	4.16 ± 0.12	2.61 ± 0.10	3.92 ± 0.09	2.45 ± 0.04	0.4605	0.5214	4	5	4	6
	HP	4.66 ± 0.11	3.57 ± 0.53	4.56 ± 0.05	2.17 ± 0.08	0.9716	0.9994	4	6	5	6
*Per2*	CTX	7.70 ± 0.23	6.34 ± 0.15	7.21 ± 0.03	5.95 ± 0.15	0.0121	0.0113	4	5	4	6
	HP	6.98 ± 0.23	4.46 ± 0.89	6.88 ± 0.11	5.05 ± 0.24	0.9716	0.6197	4	6	5	6
*Rev-Erba*	CTX	3.89 ± 0.03	2.51 ± 0.06	3.91 ± 0.02	2.47 ± 0.07	0.9536	0.9276	4	5	4	6
	HP	4.97 ± 0.07	2.34 ± 0.09	4.96 ± 0.06	2.26 ± 0.11	0.9993	0.9805	4	6	5	6
*Dbp*	CTX	6.42 ± 0.07	5.47 ± 0.10	6.35 ± 0.09	5.49 ± 0.07	0.7058	0.9276	4	5	4	6
	HP	7.62 ± 0.08	6.12 ± 0.23	7.63 ± 0.10	6.10 ± 0.25	0.9993	0.9805	4	6	5	6
*Tef*	CTX	3.98 ± 0.02	3.35 ± 0.06	3.96 ± 0.02	3.24 ± 0.05	0.9536	0.7245	4	5	4	6
	HP	4.80 ± 0.09	3.23 ± 0.05	4.80 ± 0.02	2.94 ± 0.07	0.9993	0.7872	4	6	5	6
*Nfil3*	CTX	7.11 ± 0.05	6.46 ± 0.17	6.88 ± 0.06	6.13 ± 0.07	0.0102	0.0206	4	5	4	6
	HP	7.86 ± 0.10	8.58 ± 0.47	7.71 ± 0.13	8.22 ± 0.21	0.6950	0.713	4	6	5	6
*Dec1*	CTX	4.88 ± 0.04	3.62 ± 0.09	4.66 ± 0.02	3.27 ± 0.04	0.0102	0.0143	4	5	4	6
	HP	5.54 ± 0.11	3.92 ± 0.11	5.49 ± 0.07	3.82 ± 0.07	0.9951	0.9805	4	6	5	6

To better characterize how SD affects activity-regulated pathways in Camk2a^+^ and PV^+^ populations, we quantified ribosome-associated transcript abundance for IEGs encoding transcription regulatory factors, *Npas4*, *Cfos*, and *Fosb*. We first quantified transcript abundance in Camk2a^+^ neocortical and hippocampal neuronal populations after 3 h of *ad libitum* sleep (sleep; *n* = 4) or SD (*n* = 5), starting at lights on (ZT0). Three-hour SD produced no significant change in ribosome-associated transcript abundance in Camk2a^+^ neocortical cells (*N.S*. for all transcripts, Holm–Sidak *post hoc* test) while significantly increasing *Cfos* abundance in the hippocampus (*p* < 0.05; Fig. 2*E*; Table 2). After prolonged (6 h) SD, neocortical *Npas4* (*p* < 0.01), *Cfos* (*p* < 0.0001) and *Fosb* (*p* < 0.01) abundance increased on ribosomes in Camk2a^+^ neurons. In the hippocampus, ribosome-associated *Npas4* (*p* < 0.001), *Cfos* (*p* < 0.0001), and *Fosb* (*p* < 0.0001) all increased in abundance in Camk2a^+^ neurons after 6-h SD.

We next quantified ribosome-associated transcripts encoding IEG transcription factors in PV^+^ interneurons from the neocortex (*n* = 4 mice/group) and hippocampus (*n* = 4 and *n* = 5 mice for sleep and SD; Table 3). Three-hour SD significantly increased neocortical *Npas4* and *Cfos* (*p* < 0.05) abundance, but had no effect on transcript abundance in the hippocampus (N.S. for all transcripts, Holm–Sidak *post hoc* test). Six-hour SD significantly increased all three transcripts' abundance (*p* < 0.0001 for *Cfos, p* < 0.001 for all other transcripts) in the neocortex, but only affected *Cfos* in the hippocampus (*p* < 0.01). Overall, ribosome-associated transcript abundance in PV^+^ interneurons from the neocortex underwent fold changes that were slightly higher than hippocampus.

### Subregion-specific and layer-specific effects of SD on mRNA abundance in *pvalb*+ and non-*Pvalb*+ neurons

Recent findings suggest that effects of SD on transcription and translation may be more region-specific and subregion-specific than previously thought (Delorme et al., 2019; Havekes and Aton, 2020). To more precisely characterize region-specific and cell type-specific changes in overall mRNA abundance after SD, and build on results from TRAP experiments, we used fluorescence *in situ* hybridization to visualize *Pvalb*, *Arc*, *Homer1a*, and *Cfos* transcripts in C57Bl6/J mice after 6-h SD (*n* = 6) or *ad libitum* sleep (*n* = 5; Figs. 3*A–H*, 4*A*,*B*). Transcripts were quantified separately in neocortical layers 1–6 and dorsal hippocampal areas DG, CA3, and CA1. *Pvalb* expression was used to discriminate expression in PV^+^ interneurons from that in non-PV^+^ (mainly pyramidal) neurons. ROIs for *Pvalb*+ interneurons and non-*Pvalb*+ regions were identified separately and total transcript expression (total fluorescence dot number) was calculated relative to background signal and normalized to the area of their respective ROI. We first quantified mRNA abundance after sleep versus SD among non-*Pvalb*+ cells in neocortical regions overlying dorsal hippocampus (including S1; Fig. 3*C*). Across neocortex as a whole, SD significantly increased *Arc* in non-*Pvalb*+ neurons (sleep = 24.8 ± 10.3 vs SD = 79.2 ± 10.1 dots/mm^2^, *p* < 0.05, Holm–Sidak *post hoc* test), and showed a tendency for increasing *Cfos* (sleep = 8.6 ± 3.9 vs SD = 26.2 ± 5.1 dots/mm^2^, *p* = 0.053) and *Homer1a* (sleep = 1.4 ± 0.5 vs SD = 7.8 ± 2.6 dots/mm^2^, *p* = 0.056). Expression was also quantified in individual neocortical layers. The largest effects of SD were seen for *Homer1a* and *Cfos* in layers 4 (*Homer1a*: sleep = 1.6 ± 0.6 vs SD = 7.8 ± 2.2 dots/mm^2^, *Cfos*: sleep = 13.5 ± 6.4 vs SD = 40.5 ± 7.1 dots/mm^2^) and 5 (*Homer1a*: sleep = 1.5 ± 0.4 vs SD = 9.5 2.8 dots/mm^2^, *Cfos*: sleep = 8.8 ± 3.8 vs SD = 34.5 ± 6.9 dots/mm^2^, *p* < 0.05). SD increased *Arc* dots/mm^2^ significantly across layers 2/3 (sleep = 15.2 ± 5.8 vs SD = 45.8 ± 3.7 dots/mm^2^, *p* < 0.01, unpaired *t* test), 4 (sleep = 36.3 ± 14.3 vs SD = 137.5 ± 17.7 dots/mm^2^, *p* < 0.01), and 5 (sleep = 21.7 ± 8.2 vs SD = 81.7 ± 12.8 dots/mm^2^, *p* < 0.05; Fig. 3*I*). No changes in expression were observed with SD in layer 6, and layer 1 expression was not analyzed because of low overall expression and cell density.

In dramatic contrast to the relatively large changes in IEG transcript abundance in putative pyramidal cells in neocortex following SD, neither *Arc* nor *Homer1a* (N.S., Holm–Sidak *post hoc* test) levels were significantly altered by SD in non-*Pvalb*+ cells in any region of dorsal hippocampus (Fig. 4*C*). *Cfos* was increased significantly in non-*Pvalb*+ cells with SD in CA3 only (sleep = 2.8 ± 0.5 vs SD = 10.7 ± 1.4 dots/mm^2^, *p* < 0.01) with no significant changes in CA1 or DG (*N.S.*, Student's *t* test). This differs from findings using TRAP (Fig. 2*D*,*E*), where all three transcripts were increased on translating ribosomes from both the neocortex and hippocampus of *Camk2a::RiboTag* mice after 6-h SD.

We then quantified transcript abundance within PV^+^ interneurons, using *Pvalb* mRNA expression to define the *Pvalb*+ ROI (Fig. 3*C–F*). Overall, IEG expression in *Pvalb*+ cells was relatively low. SD caused no significant changes in *Arc* or *Homer1a* in any layer of the neocortex, although *Cfos* dots/µm^2^ increased selectively in *Pvalb*+ cells in layer 2/3 (sleep = 0.014 ± 0.002 vs SD = 0.043 ± 0.009 dots/µm^2^, *p* < 0.01; Fig. 3*J*). Because many *Pvalb*+ cells expressed no detectable IEGs, we also quantified expression within the subpopulation of *Pvalb*+ interneurons which had detectable levels of mRNA expression. Using a semi-automated protocol for this more circumscribed analysis, we found that SD did not affect expression levels for *Arc* or *Cfos*, but did increase *Homer1a* dots/µm^2^ when measured across the entire neocortex (Fig. 3*K*). Thus, in contrast to significant increases in ribosome-associated transcripts in neocortical PV^+^ interneurons observed after 6-h SD (Fig. 2*D*,*E*), changes in total transcript levels in these cells (measured with *in situ* hybridization) were relatively minimal. Consistent with the generally limited ribosome-associated transcript changes observed in hippocampus with SD (Fig. 2*D*,*E*), no significant changes in IEG expression were observed in *Pvalb*+ interneurons from any region of dorsal hippocampus with SD, regardless of method for quantification (Fig. 4*D*,*E*).

One possibility is that the relative proportion of IEG^+^ PV^+^ interneurons varied as a function of SD. Because *Pvalb*+ interneurons varied substantially in terms of ROI size, we quantified the IEG+ proportion of *Pvalb*+ interneurons in sleep and SD mice, as a function of both cell count and ROI area (Fig. 5). We found that SD significantly increased the proportion of *Arc*+ and *Cfos*+ *Pvalb*+ interneurons in the neocortex, across all layers quantified (Fig. 5*B*). No significant differences were observed in the proportion of *Homer1a*+ *Pvalb*+ interneurons. Similarly, we found significant increases in the proportion of *Arc*+ and *Cfos*+ *Pvalb*+ area after SD for all neocortical layers, with the exception of layer 5. No differences were observed for *Homer1a*+ area with PV^+^ interneurons using this measure. No significant changes in any of the mRNAs' expression were observed after SD in *Pvalb*+ interneurons in any region of the hippocampus after SD, regardless of the method of quantification (Fig. 5*C*).

Critically, *Pvalb* expression itself can be regulated as a function of synaptic plasticity (Donato et al., 2013). We found that when expression values were calculated cell by cell, *Pvalb* levels did vary in both DG and neocortex as a function of SD (values plotted as cumulative distributions in Fig. 5*D*). These changes moved in opposite directions, with DG neurons showing SD-driven decreases in *Pvalb* labeling intensity, and neocortex showing SD-driven increases in *Pvalb*.

Together, our *in situ* hybridization data suggest that SD: (1) drives relatively modest changes in *Homer1a*, *Arc*, and *Cfos* in neocortical PV^+^ interneurons; (2) does not affect these transcripts in hippocampal PV^+^ interneurons; and (3) drives differential changes in expression of *Pvalb* expression in hippocampal versus neocortical PV^+^ interneurons.

### Cell type-specific and region-specific effects of SD on ribosome-associated transcripts involved in circadian timekeeping

**Figure 6.**
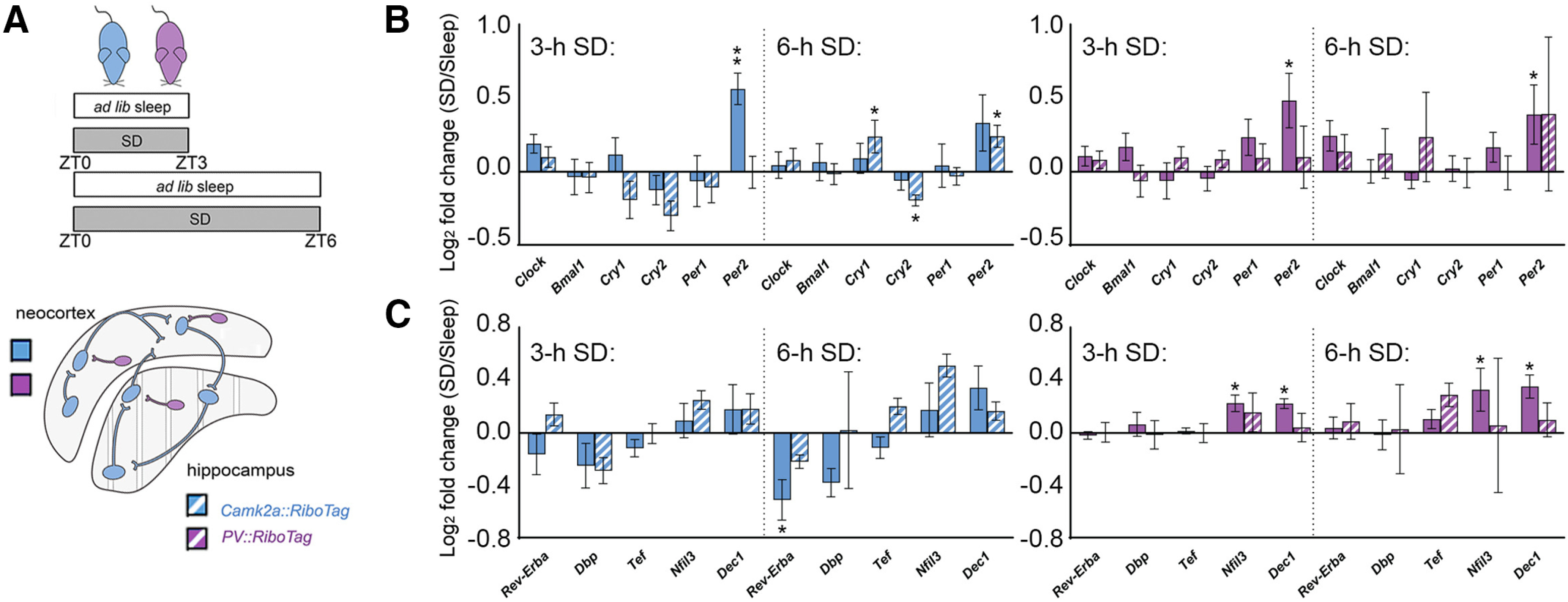
SD alters ribosome-associated transcripts encoding core clock genes and circadian clock modifiers in a cell type-specific and region-specific manner. ***A***, Three- and 6-h SD effects on ribosome-associated transcript abundance were quantified separately from neocortex and hippocampus from *Camk2a::RiboTag* (blue) and *PV::RiboTag* (violet) transgenic mice. ***B***, Three-hour SD significantly increased *Per2* abundance on ribosomes in Camk2a^+^ (blue) and PV^+^ (magenta) neocortical neurons; no significant changes in core clock transcripts were observed in hippocampal neurons. After 6-h SD, *Per2* abundance remained significantly elevated in neocortical PV^+^ interneurons. Ribosome-associated *Cry1*, *Cry2*, and *Per2* were all altered after 6-h SD in the hippocampal Camk2a^+^ neuron population. No significant change observed among PV^+^ interneurons. ***C***, Three-hour SD had no significant effect on ribosome-associated circadian clock modifier transcripts among Camk2a^+^ (blue) neurons in neocortex, but increased *Nfil3* and *Dec1* expression among neocortical PV^+^ interneurons (magenta). Six-hour SD significantly decreased *Rev-Erb*α abundance on ribosomes in Camk2a^+^ neocortical neurons. No transcripts were significantly altered by SD in either neuron population in hippocampus. Transcript level changes are presented as a log_2_ fold change between SD and *ad libitum* sleep mice. Values indicate mean ± SEM with propagated error; *p < 0.05, **p < 0.01, Holm–Sidak *post hoc* test versus sleep.

SD has previously been implicated in regulating core molecular clock genes' expression (Bolsius et al., 2021). This effect of SD may be similar to regulation of IEG expression, particularly as clock genes such as *Per1* and *Per2* can be regulated by cellular activity in a manner similar to other IEGs (Balsalobre et al., 1998; Kuhlman et al., 2003; Lee et al., 2010). However, similar to other IEGs, the extent to which SD differentially impacts core clock gene expression as a function of cell type and brain region is unclear. Consequently, we quantified ribosome-associated transcript abundance for core clock genes, *Clock*, *Per1*, *Per2*, *Cry1*, *Cry1*, and *Bmal1*, after SD in Camk2a^+^ neurons and PV^+^ interneurons of the neocortex and hippocampus (Fig. 6*A*). Consistent with findings from whole neocortical tissue (Franken et al., 2007; Hoekstra et al., 2019), we found that 3-h SD significantly increased *Per2* expression in neocortical Camk2a^+^ neurons and PV^+^ interneurons (Fig. 6*B*). In contrast, SD had no significant impact on transcript abundance in the hippocampus of either population. Longer-duration (6 h) SD resulted in no further changes in neocortical transcript abundance (with *Per2* levels tending to remain elevated in both Camk2a^+^ neurons and PV^+^ interneurons). Within the hippocampus, 6-h SD significantly altered abundance of ribosome-associated *Per2*, *Cry1*, and *Cry2* transcripts in Camk2a^+^ neurons (increasing *Per2* and *Cry1*, decreasing *Cry2*), while having no significant effect on transcript abundance in PV^+^ interneurons.

We also quantified (after SD vs sleep) the abundance of ribosome-associated mRNAs encoding other cellular timekeeping components: *Rev-Erb*α, *Dbp*, *Ted*, *Nfil3*, and *Dec1* (Fig. 6*C*). We found significant heterogeneity in how these auxiliary clock genes responded to SD in different cell types and regions. None of the transcripts were significantly altered in either cell type in the hippocampus, with either 3- or 6-h SD. However, within the neocortex, both 3- and 6-h SD significantly increased cortical *Nfil3* and *Dec1* abundance in PV^+^ interneurons. While these transcripts were not significantly altered in neocortical Camk2a^+^ neurons, 6-h SD significantly decreased *Rev-Erb*α expression in Camk2a^+^ neocortical neurons. Together, these data suggest that mRNAs associated with cellular timekeeping are differentially affected, based on brain region and cell type.

## Discussion

Here, using TRAP, we have identified SD-driven molecular changes unique to specific cell populations in hippocampus and neocortex. Numerous studies have used transcriptome (Cirelli et al., 2004; Vecsey et al., 2012) or proteome (Poirrier et al., 2008; Noya et al., 2019) profiling of these structures following sleep versus SD as a way of clarifying the functions of sleep in the brain. We find that comparing across structures, there are large differences in SD effects on ribosome-associated transcripts. For example, while even brief (3 h) SD increases abundance of plasticity-mediating transcripts in neocortical Camk2a^+^ neurons and PV^+^ interneurons (Fig. 2*D*), few of these transcripts are altered in hippocampus even after longer SD. This is particularly true for hippocampal PV^+^ interneurons, for which none of the transcripts are significantly altered by SD SD-driven changes in abundance for IEG transcription regulators follow a similar pattern (Fig. 2*E*), with hippocampal PV^+^ interneurons in particular being refractory to SD. Our *in situ* analysis of mRNA abundance in *Pvalb*+ and *Pvalb*- neurons (Figs. 3–5) is consistent with this interpretation, and suggests that even within neocortex, SD-driven changes in these transcripts' abundance are relatively modest in PV^+^ interneurons.

Critically, our TRAP data and *in situ* hybridization data generally suggested similar effects of SD on mRNA abundance (i.e., comparing Figs. 2 and 3, 4). Thus, in general, while our current TRAP findings cannot differentiate SD-mediated transcriptional and translational effects, a parsimonious interpretation is that SD differentially drives transcription of many genes in excitatory and inhibitory neurons. However, for some mRNA species, SD-driven increases in IEG expression for a given cell type and structure were observed using TRAP, but were not seen with *in situ*. At present, we cannot rule out technical differences (e.g., lower sensitivity for transcript detection using *in situ* compared with TRAP) as an underlying cause of this discrepancy. Another possibility is that the specific brain areas measured using *in situ* (dorsal hippocampus and somatosensory and posterior parietal cortex) are selectively less sensitive to SD than other hippocampal and neocortical regions (which would contribute only to TRAP-based profiling). However, this seems unlikely, as sensory input during SD would likely drive activity higher in those particular areas (Havekes and Aton, 2020). A final, highly speculative possibility is that SD may differentially affect transcription and translation rates of specific mRNAs in some brain areas and cell types. For example, if transcription rates were unaffected, but mRNAs differentially associated with ribosomes, this could lead to differences between TRAP and *in situ* results we see after SD for IEGs in neocortical PV^+^ interneurons, or for most hippocampal neurons.

While IEGs are generally assumed to reflect specific patterns of recent neuronal activity (Tyssowski and Gray, 2019), there are brain region-specific and microcircuit-specific differences in IEG expression which reflect neurons' network connectivity patterns (Tyssowski et al., 2018; Gonzales et al., 2020). Moreover, IEG expression in PV^+^ interneurons is regulated by distinct cellular pathways and is differentially gated by neuronal activation (Cohen et al., 2016). Indeed, some studies have failed to detect IEGs in PV^+^ interneurons altogether (Vazdarjanova et al., 2006; Imamura et al., 2011), and our present results showing relatively low expression in the *Pvalb*+ interneuron population (Figs. 3–5). However, insofar as abundance of all of these transcripts is likely regulated by neuronal activity to some degree (Donato et al., 2013; Yap and Greenberg, 2018), our present data support two broad conclusions. First, while SD seems to increase neuronal activation (and IEG expression) across neocortex, these effects are less pronounced in the hippocampus. Second, PV^+^ interneuron activity (and IEG expression) may vary less as a function of SD than Camk2a^+^ neuron activity.

The former conclusion has major implications for the field of learning and memory, where pronounced and selective effects of sleep disruption on hippocampal processes (e.g., episodic and spatial memory consolidation) have been well described (Saletin and Walker, 2012; Havekes and Abel, 2017; Delorme et al., 2019; Puentes-Mestril et al., 2019). Human brain imaging has shown that SD can lead to decreased capacity for hippocampal circuit activation during memory encoding or recall (Yoo et al., 2007). In hippocampal structures such as the DG and CA1, available data suggest that both markers of neuronal activity and synaptic plasticity are disrupted after SD (Havekes et al., 2016; Tudor et al., 2016; Ognjanovski et al., 2018; Delorme et al., 2019; Raven et al., 2019). Our present data largely confirm these findings, and suggest that particularly in dorsal hippocampal DG and CA1 (Fig. 4), there is little evidence of neuronal activity levels (and IEG expression) increasing across a period of SD. Indeed, we find that DG neurons show decreased *Pvalb* expression after SD, while neocortical neurons simultaneously show increased expression (Fig. 5). Critically, *Pvalb* expression levels have been shown to correlate with both PV^+^ interneuron activity level and the relative amounts of excitatory to inhibitory input PV^+^ interneurons receive (Donato et al., 2013). With this fact in mind, it is plausible that SD increases excitatory input to PV^+^ interneurons in neocortex, while simultaneously decreasing excitatory input to PV^+^ interneurons in DG. Such an effect of SD on the DG parallels our recent work showing differential effects of SD on another activity marker, *Arc*, in DG versus neocortex, and suggests that SD may have a uniquely disruptive effect on network activity in DG.

The latter conclusion also has important implications for maintenance of E-I balance during SD. Recent data suggest that E-I balance normally varies over the course of the day, in a sleep-dependent manner (Bridi et al., 2020). Furthermore, prior evidence from both whole-tissue transcriptome profiling and immunohistochemistry has suggested that SD may differentially affect connections from excitatory to inhibitory neurons (and vice versa) in structures like the neocortex (Del Cid-Pellitero et al., 2017; Puentes-Mestril and Aton, 2017). Because sleep loss is one of the major risk factors for triggering seizure onset in epilepsy (Frucht et al., 2000; Lawn et al., 2014), an underlying mechanism might be differential activation of, or plasticity in, interneurons versus principal neurons with SD. Interactions between PV^+^ interneurons and principal neurons are particularly important in both regulation of attention (Aton, 2013) and in generating network oscillations important for memory consolidation (Ognjanovski et al., 2017, 2018). Insofar as SD may disrupt both attention and memory consolidation, differential effects on activity of PV^+^ and Camk2a^+^ neurons in the hippocampus and neocortex may be an important underlying mechanism.

Because many of the transcripts quantified here (e.g., *Arc*, *Homer1a*, *Narp*, and *Bdnf*) play a critical role in activity-regulated synaptic plasticity, the fact that their abundance in Camk2+ and PV^+^ neurons is differentially altered by SD also has intriguing implications. For example, it suggests that SD could lead to long-lasting changes in the E-I balance and information processing capacity of neocortical and hippocampal circuits. This may be a plausible mechanism for some of the reported longer-lasting brain metabolic (Wu et al., 2006) and cognitive (Dinges et al., 1997; Belenky et al., 2003; Chai et al., 2020) effects of SD (i.e., those that do not normalize with recovery sleep).

Alterations in brain clock gene expression with SD has been widely reported (Wisor et al., 2002, 2008; Franken et al., 2007; Mongrain et al., 2011; Bolsius et al., 2021). Along with transcripts such as *Homer1a* (Maret et al., 2007; Zhu et al., 2020), SD-driven increases in clock transcripts such as *Per2* are hypothesized to (1) act as an immediate-early gene response (similar to increases in *Arc* and *Cfos*; Balsalobre et al., 1998; Kuhlman et al., 2003; Lee et al., 2010) and (2) play a role in homeostatic aspects of sleep regulation (Franken et al., 2007; Mang and Franken, 2015). Our data suggest that similar to plasticity-regulating transcripts (including *Homer1a*), SD-mediated changes in clock gene transcripts on ribosomes are cell type specific and brain region specific (Fig. 6). For example, while *Per2* increases on both Camk2a^+^ and PV^+^ neocortical neuron-derived ribosomes with as little as 3-h SD, no clock gene transcripts are altered in the hippocampus with 3-h SD. Another example is *Rev-erb*α, which is significantly reduced after 6-h SD, but only in neocortical Camk2a^+^ neurons. An interesting and important issue, raised by our findings, is that SD-driven changes in particular core clock transcripts' abundance do not move in the same direction, as they normally would during a 24 h cycle (e.g., *Cry1*, *Cry2*, and *Per2*). This suggests that SD-driven changes in these transcripts are not likely driven by canonical E-box elements, consistent with recent findings (Mongrain et al., 2011). However, because changes in these transcripts may have numerous downstream effects on transcription of other clock-control genes (Schmutz et al., 2010; Chiou et al., 2016), these SD-driven changes may have even more numerous downstream effects that changes in plasticity effectors' transcripts. Future studies will be needed to quantify longer-term cell type-specific changes to physiology and structure initiated during SD, and the molecular events responsible for these changes.

Together, our data suggest that effects of SD on plasticity, timekeeping, and homeostatic regulation of brain circuitry is heterogeneous, and likely involves subtle modifications to microcircuits (e.g., those in hippocampal subregions and neocortical layers) critical for appropriate brain function.
