# Supplementary material for: Sleep Loss Drives Brain Region-Specific and Cell Type-Specific Alterations in Ribosome-Associated Transcripts Involved in Synaptic Plasticity and Cellular Timekeeping
Source: J Neurosci. 2021 Jun 23;41(25):5386–98. doi: 10.1523/JNEUROSCI.1883-20.2021 (PMC8221591; doi:10.1523/JNEUROSCI.1883-20.2021)
Supplement: Extended Data Table 1-1. — Primer sequences for qPCR. Download Table 1-1, DOCX file. [file ns-JN-RM-1883-20-s01.docx]

| Gene Name | Forward Primer | Reverse Primer |
| --- | --- | --- |
| *Actg1* | ACTCTTCCAGCCTTCCTTC | ATCTCCTTCTGCATCCTGTC |
| *Hprt* | CGTGATTAGCGATGATGAACCA | CTTTCAGTCCTGTCCATAATCAGT |
| *Gapdh* | GTGTTTCCTCGTCCCGTAGA | AATCCGTTCACACCGACCTT |
| *Pgk1* | TCGTGATGAGGGTGGACTTC | ACAGCAGCCTTGATCCTTTG |
| *Cypa* | CCACCGTGTTCTTCGACATC | AGGAACCCTTATAGCCAAATCCT |
| *Tuba4a* | ATGCGCGAGTGCATTTCAG | CACCAATGGTCTTATCGCTGG |
| *Tbp* | GCAGCCTCAGTACAGCAATC | GGTGCAGTGGTCAGAGTTTG |
| *Arc* | CCAGATCCAGAACCACATGAA | GAGAGTGTACCCTCACTGTATTG |
| *Homer1a* | GCATTGCCATTTCCACATAGG | ATGAACTTCCATATTTATCCACCTTACTT |
| *Narp* | TGCTGATAGAGTGGGGCAAT | CAGCTGTGCGACCTTGTC |
| *Bdnf* | GGTCACAGCGGCAGATAAA | TCAGTTGGCCTTTGGATACC |
| *Npas4* | CTTCTCAACACTACCGCCTG | TGCTTGGCTTGAAGTCTCAC |
| *cFos* | GAAGAGGAAGAGAAACGGAGAAT | CTTGGAGTGTATCTGTCAGCTC |
| *FosB* | AGAAGACCCCGAGAAGAGAC | TCTTCGTAGGGGATCTTGCA |
| *Clock* | CCAAAGGCCAGCAGTGGATA | TTGTCAGCAGCTGTCTCAGG |
| *Bmal1* | CCCATACACAGAAGCAAACT | ACAGACTCGGAGACAAAGAG |
| *Cry1* | TCCCCTCCCCTTTCTCTTTA | TTGTCCCAAGGGATCTGAAC |
| *Cry2* | AAGCTGAATTCGCGTCTGTT | AACAGCCTTGGGAACACATC |
| *Per1* | CCAGGATGTGGGTGTCTTCT | TTTCCTGGGTGAAGTCCTTG |
| *Per2* | AAGAACGCGGATATGTTTGC | CAGGATCTTCCCAGAAACCA |
| *Rev-Erba* | CGACCCTGGACTCCAATAACA | AACCTTGAGTCAGGGACTGG |
| *Dbp* | GCCAGCTGCTTGACATCTAGG | GCATCTCTCGACCTCTTGGC |
| *Tef* | CCTTCCCTCTGGTCCTGAAGA | CAGAGACGGCCATGGTACTG |
| *Nfil3* | GAGGGTGTAGTGGGCAAGTC | ATCCGAAGCTTGTGCGGTAA |
| *Dec1* | TGGCGAAGCATGAGAACACT | CTTTGGGAGCCGAGTCCAAT |
| *Mbp* | CCTTGACTCCATCGGGCGCT | CTTCTGGGGCAGGGAGCCAT |
| *Gfap* | TCCTGGAACAGCAAAACAAG | CAGCCTCAGGTTGGTTTCAT |
| *NPY* | CAAGAGATCCAGCCCTGAGA | ACATGGAAGGGTCTTCAAGC |
| *SOM* | CTCGGACCCCAGACTCCGTC | CTCGGGCTCCAGGGCATCAT |
| *Griar4* | GTTTTCCCTGGGTGCCTTTA | GAAGAACCACCATACGCCTC |
| *Gad67* | GACACCGGGGACAAGGCGAT | TCCCACGGTGCCCTTTGCTT |
| *Parvalbumin* | GTCGATGACAGACGTGCTCA | TTGTGGTCGAAGGAGTCTGC |
| *Vglut2* | ATACTAGAGGGGTGGCCATC | GTGCAGCAATGAGGAAGACA |
| *Vglut1* | CCAGCATCTCTGAGGAGGAG | GGCTGAGAGATGAGGAGCAG |
| *Camk2a* | GCCTGTACCAGCAGATCATCAAA | GGGTTGATGGTCAGCATCTTA |

**Extended Data Table 1-1.** Primer sequences for qPCR

**Table S1.** Primer Designs
