# Supplementary material for: Sleep Loss Drives Brain Region-Specific and Cell Type-Specific Alterations in Ribosome-Associated Transcripts Involved in Synaptic Plasticity and Cellular Timekeeping
Source: J Neurosci. 2021 Jun 23;41(25):5386–98. doi: 10.1523/JNEUROSCI.1883-20.2021 (PMC8221591; doi:10.1523/JNEUROSCI.1883-20.2021)
Supplement: Extended Data Table 1-2. — Housekeeping gene stability analysis for Camk2a::RiboTag qPCR. Methods for stability analysis are described in Materials and Methods. *Genorm automatically calculates the stability measure for the two most stable genes. Download Table 1-2, DOCX file. [file ns-JN-RM-1883-20-s02.docx]

**Extended Data Table 1-2.** Housekeeping gene stability analysis for *Camk2a::RiboTag* qPCR. *Genorm automatically calculates the stability measure for the two most stable genes.

|  |  |  | Comprehensive | | Genorm* | | NormFinder | | Intergroup Variation | | Intragroup Variation | |
| --- | --- | --- | --- | --- | --- | --- | --- | --- | --- | --- | --- | --- |
| Gene name | Condition | Region | Stability | Ranking | Stability | Ranking | Stability | Ranking | S | SD | S | SD |
| *Actg1* | 3-h | CTX | 4.95 | 6 | 0.066 | 3 | 0.025 | 6 | -0.005 | 0.005 | 0.003 | 0.003 |
|  |  | HIP | 5.18 | 6 | 0.112 | 5 | 0.060 | 4 | 0.026 | -0.026 | 0.003 | 0.005 |
|  | 6-h | CTX | 7 | 7 | 0.158 | 6 | 0.102 | 7 | 0.066 | -0.066 | 0.038 | 0.005 |
|  |  | HIP | 7 | 7 | 0.147 | 6 | 0.053 | 7 | 0.010 | -0.010 | 0.020 | 0.014 |
| *Hprt* | 3-h | CTX | 2.45 | 2 | 0.057 | 2 | 0.012 | 2 | 0.009 | -0.009 | 0.000 | 0.002 |
|  |  | HIP | 7 | 7 | 0.183 | 6 | 0.151 | 6 | -0.119 | 0.119 | 0.085 | 0.011 |
|  | 6-h | CTX | 2.66 | 3 | 0.081 | 1 | 0.061 | 4 | -0.028 | 0.028 | 0.009 | 0.002 |
|  |  | HIP | 1 | 1 | 0.055 | 1 | 0.012 | 1 | 0.013 | -0.013 | 0.000 | 0.002 |
| *Gapdh* | 3-h | CTX | 1.41 | 1 | 0.048 | 1 | 0.009 | 1 | 0.010 | -0.010 | 0.000 | 0.000 |
|  |  | HIP | 1 | 1 | 0.049 | 1 | 0.037 | 1 | 0.012 | -0.012 | 0.002 | 0.002 |
|  | 6-h | CTX | 6 | 6 | 0.127 | 5 | 0.078 | 6 | 0.060 | -0.060 | 0.003 | 0.001 |
|  |  | HIP | 4.56 | 5 | 0.126 | 5 | 0.037 | 6 | 0.013 | -0.013 | 0.008 | 0.009 |
| *Pgk1* | 3-h | CTX | 2.82 | 3 | 0.048 | 1 | 0.020 | 4 | 0.006 | -0.006 | 0.002 | 0.001 |
|  |  | HIP | 3.46 | 4 | 0.060 | 2 | 0.069 | 5 | 0.025 | -0.025 | 0.011 | 0.004 |
|  | 6-h | CTX | 1.73 | 1 | 0.081 | 1 | 0.052 | 2 | -0.016 | 0.016 | 0.009 | 0.003 |
|  |  | HIP | 5.23 | 6 | 0.111 | 4 | 0.031 | 5 | 0.003 | -0.003 | 0.004 | 0.007 |
| *Cypa* | 3-h | CTX | 3.56 | 4 | 0.072 | 4 | 0.019 | 3 | 0.015 | -0.015 | 0.001 | 0.002 |
|  |  | HIP | 3.22 | 3 | 0.084 | 3 | 0.052 | 3 | 0.030 | -0.030 | 0.000 | 0.004 |
|  | 6-h | CTX | 3.46 | 5 | 0.088 | 2 | 0.067 | 5 | -0.038 | 0.038 | 0.006 | 0.003 |
|  |  | HIP | 2 | 2 | 0.055 | 6 | 0.021 | 3 | 0.009 | -0.009 | 0.005 | 0.001 |
| *Tuba4a* | 3-h | CTX | 5.73 | 7 | 0.081 | 5 | 0.022 | 5 | -0.025 | 0.025 | 0.002 | 0.002 |
|  |  | HIP | 2.11 | 2 | 0.049 | 1 | 0.045 | 2 | 0.013 | -0.013 | 0.004 | 0.002 |
|  | 6-h | CTX | 2.24 | 2 | 0.105 | 4 | 0.022 | 1 | 0.000 | 0.000 | 0.000 | 0.003 |
|  |  | HIP | 4.33 | 4 | 0.089 | 3 | 0.024 | 4 | -0.018 | 0.018 | 0.001 | 0.007 |
| *Tbp* | 3-h | CTX | 4.3 | 5 | 0.085 | 6 | 0.027 | 7 | -0.011 | 0.011 | 0.003 | 0.003 |
|  |  | HIP | 4.95 | 5 | 0.099 | 4 | 0.045 | 2 | 0.013 | -0.013 | 0.001 | 0.006 |
|  | 6-h | CTX | 2.83 | 4 | 0.096 | 3 | 0.060 | 3 | -0.044 | 0.044 | 0.001 | 0.001 |
|  |  | HIP | 3 | 3 | 0.078 | 2 | 0.020 | 2 | -0.030 | 0.030 | 0.003 | 0.002 |
