# Supplementary material for: Sleep Loss Drives Brain Region-Specific and Cell Type-Specific Alterations in Ribosome-Associated Transcripts Involved in Synaptic Plasticity and Cellular Timekeeping
Source: J Neurosci. 2021 Jun 23;41(25):5386–98. doi: 10.1523/JNEUROSCI.1883-20.2021 (PMC8221591; doi:10.1523/JNEUROSCI.1883-20.2021)
Supplement: Extended Data Table 1-3. — Housekeeping gene stability analysis for PV::RiboTag qPCR. *Genorm automatically calculates the stability measure for the two most stable genes. Download Table 1-3, DOCX file. [file ns-JN-RM-1883-20-s03.docx]

**Extended Data Table 1-3.** Housekeeping gene stability analysis for *PV::RiboTag* qPCR. *Genorm automatically calculates the stability measure for the two most stable genes.

|  |  |  | Comprehensive | | Genorm* | | NormFinder | | Intergroup Variation | | Intragroup Variation | |
| --- | --- | --- | --- | --- | --- | --- | --- | --- | --- | --- | --- | --- |
| Gene name | Condition | Region | Stability | Ranking | Stability | Ranking | Stability | Ranking | S | SD | S | SD |
| *Actg1* | 3-h | CTX | 5.12 | 5 | 0.089 | 6 | 0.033 | 5 | -0.007 | 0.007 | 0.003 | 0.003 |
|  |  | HIP | 3.76 | 5 | 0.189 | 5 | 0.046 | 4 | -0.032 | 0.032 | 0.028 | 0.001 |
|  | 6-h | CTX | 7 | 7 | 0.142 | 6 | 0.063 | 7 | -0.032 | 0.032 | 0.019 | 0.010 |
|  |  | HIP | 3.56 | 4 | 0.144 | 5 | 0.033 | 3 | 0.025 | -0.025 | 0.003 | 0.012 |
| *Hprt* | 3-h | CTX | 5.44 | 6 | 0.080 | 4 | 0.035 | 7 | 0.030 | -0.030 | 0.000 | 0.001 |
|  |  | HIP | 6.74 | 7 | 0.246 | 7 | 0.100 | 7 | -0.046 | 0.046 | 0.031 | 0.059 |
|  | 6-h | CTX | 1.19 | 1 | 0.077 | 1 | 0.033 | 3 | 0.020 | -0.020 | 0.002 | 0.001 |
|  |  | HIP | 1.57 | 1 | 0.106 | 1 | 0.030 | 2 | 0.022 | -0.022 | 0.005 | 0.005 |
| *Gapdh* | 3-h | CTX | 1.68 | 1 | 0.041 | 1 | 0.032 | 4 | -0.020 | 0.020 | 0.001 | 0.002 |
|  |  | HIP | 3.46 | 4 | 0.166 | 4 | 0.040 | 3 | -0.015 | 0.015 | 0.010 | 0.005 |
|  | 6-h | CTX | 2.51 | 3 | 0.099 | 3 | 0.032 | 2 | 0.023 | -0.023 | 0.001 | 0.001 |
|  |  | HIP | 2.34 | 3 | 0.106 | 1 | 0.019 | 1 | 0.039 | -0.039 | 0.000 | 0.008 |
| *Pgk1* | 3-h | CTX | 2.28 | 3 | 0.041 | 1 | 0.031 | 3 | -0.019 | 0.019 | 0.001 | 0.003 |
|  |  | HIP | 1.32 | 1 | 0.143 | 1 | 0.014 | 1 | -0.015 | 0.015 | 0.002 | 0.000 |
|  | 6-h | CTX | 2.28 | 2 | 0.077 | 1 | 0.031 | 1 | -0.001 | 0.001 | 0.008 | 0.002 |
|  |  | HIP | 4.16 | 5 | 0.135 | 3 | 0.042 | 4 | -0.012 | 0.012 | 0.016 | 0.007 |
| *Cypa* | 3-h | CTX | 6 | 7 | 0.085 | 5 | 0.034 | 6 | 0.021 | -0.021 | 0.002 | 0.003 |
|  |  | HIP | 6.24 | 6 | 0.215 | 5 | 0.068 | 6 | 0.027 | -0.027 | 0.056 | 0.004 |
|  | 6-h | CTX | 4.56 | 5 | 0.12 | 5 | 0.045 | 5 | -0.025 | 0.025 | 0.003 | 0.009 |
|  |  | HIP | 7 | 7 | 0.242 | 6 | 0.085 | 6 | -0.070 | 0.070 | 0.002 | 0.138 |
| *Tuba4a* | 3-h | CTX | 1.86 | 2 | 0.059 | 2 | 0.016 | 1 | -0.003 | 0.003 | 0.000 | 0.000 |
|  |  | HIP | 2.78 | 3 | 0.152 | 2 | 0.061 | 5 | 0.045 | -0.045 | 0.010 | 0.024 |
|  | 6-h | CTX | 5.23 | 6 | 0.112 | 4 | 0.047 | 6 | 0.036 | -0.036 | 0.004 | 0.006 |
|  |  | HIP | 6 | 6 | 0.177 | 5 | 0.052 | 5 | -0.008 | 0.008 | 0.043 | 0.002 |
| *Tbp* | 3-h | CTX | 3.56 | 4 | 0.07 | 3 | 0.025 | 2 | -0.003 | 0.003 | 0.005 | 0.000 |
|  |  | HIP | 1.86 | 2 | 0.143 | 1 | 0.019 | 1 | 0.004 | -0.004 | 0.012 | 0.000 |
|  | 6-h | CTX | 3.72 | 4 | 0.091 | 2 | 0.039 | 4 | -0.020 | 0.020 | 0.002 | 0.005 |
|  |  | HIP | 1.86 | 2 | 0.124 | 2 | 0.019 | 1 | 0.004 | -0.004 | 0.003 | 0.001 |
